# Bone morphogenetic protein signaling is required for RAD51-mediated maintenance of genome integrity in vascular endothelial cells

**DOI:** 10.1038/s42003-018-0152-1

**Published:** 2018-09-24

**Authors:** Sanna Vattulainen-Collanus, Mark Southwood, Xu Dong Yang, Stephen Moore, Prajakta Ghatpande, Nicholas W. Morrell, Giorgio Lagna, Akiko Hata

**Affiliations:** 1 Cardiovascular Research Institute, University of California, San Francisco, San Francisco, 94143 CA USA; 2 Department of Pathology, Papworth Hospital, Papworth Everad, Cambridge, CB23 3RE UK; 3 Department of Medicine, University of Cambridge, Addenbrook’s Hospital, Cambridge, CB2 0QQ UK; 4 Department of Biochemistry and Biophysics, University of California, San Francisco, San Francisco, 94143 CA USA

## Abstract

The integrity of blood vessels is fundamental to vascular homeostasis. Inactivating mutations in the bone morphogenetic protein (BMP) receptor type II (BMPR2) gene cause hereditary vascular disorders, including pulmonary arterial hypertension and hereditary hemorrhagic telangiectasia, suggesting that BMPR2 and its downstream signaling pathway are pivotal to the maintenance of vascular integrity through an unknown molecular mechanism. Here we report that inactivation of BMPR2 in pulmonary vascular endothelial cells results in a deficit of RAD51, an enzyme essential for DNA repair and replication. Loss of RAD51, which causes DNA damage and cell death, is also detected in animal models and human patients with pulmonary arterial hypertension. Restoration of BMPR2 or activation of the BMP signaling pathway rescues RAD51 and prevents DNA damage. This is an unexpected role of BMP signaling in preventing the accumulation of DNA damage and the concomitant loss of endothelial integrity and vascular remodeling associated with vascular disorders.

## Abstract (web summary)

Sanna Vattulainen-Collanus et al. report that mutations in the *BMPR2* gene, which is associated with pulmonary arterial hypertension, result in a deficit of RAD51 and altered DNA repair and replication. They were able to rescue the RAD51-deficient phenotype by restoring BMPR2 activity in cell culture.

## Introduction

Bone morphogenetic proteins (BMPs) are members of the transforming growth factor-β superfamily of cytokines; they have pleiotropic activities, including regulation of cell proliferation, differentiation, and survival during embryogenic development and in adult tissues^1^. Bone morphogenetic protein signaling is mediated by heteromeric serine/threonine kinases named BMP type I and type II receptors^1^. In complex with type I BMP receptors, BMP receptor type II (BMPR2) plays an essential role in development and in maintenance of vascular homeostasis^2^. Loss-of-function mutations in the *BMPR2* gene cause severe vascular diseases, such as pulmonary arterial hypertension and, in rare cases, hereditary hemorrhagic telangiectasia^3,4^. Pulmonary arterial hypertension is a serious pulmonary vascular condition with no cure and 5-year survival rate of ~65.4%^5^. The disease is characterized by sustained elevation of vascular resistance in distal pulmonary arteries and increased pulmonary artery pressure, leading to right ventricular heart failure^5^. Up to 75% of patients with a family history of pulmonary arterial hypertension and ~20% of patients with sporadic idiopathic pulmonary arterial hypertension carry a loss-of-function mutation in the *BMPR2* gene^6^. Even pulmonary arterial hypertension patients without *BMPR2* mutations often exhibit a reduced expression of BMPR2^7^. Despite the causal link between pulmonary arterial hypertension and impairment of BMPR2 signaling^6^, the molecular etiology of pulmonary arterial hypertension remains incompletely understood. For example, in addition to genetic causes, exposure to drugs such as amphetamines, anorexigens, and chemotherapeutic agents can trigger pulmonary arterial hypertension, albeit rarely^8–10^.

Normal pulmonary vascular homeostasis is maintained by a balance between vascular repair and injury induced by various factors, such as shear stress, oxidative stress, and cellular metabolic products, including reactive oxidative species, inflammatory cytokines, and environmental toxins^11^. Endothelial cells, which line the interior surface of blood vessels in a single layer, are directly exposed to these harmful factors and are prone to injury and subsequent repair. When endothelial cells are damaged, endothelial integrity depends on the extent of the damage and the endothelial cell capacity to repair the damage^11^. Unrepaired DNA damage results in genetic mutations, recombination, premature apoptosis, and chromosomal aberrations^12^. Interestingly, endothelial cells derived from the vascular lesions of pulmonary arterial hypertension patients have been shown to be hyper-proliferative, apoptosis resistant, and genetically unstable, with microsatellite instability and mutations in genes controlling proliferation and apoptosis^13^. Likewise, somatic genomic abnormalities have been identified in the vascular lesions of pulmonary arterial hypertension patients and endothelial cells from the pulmonary arteries of pulmonary arterial hypertension patients show severe somatic chromosomal abnormalities^14^. However, it is still uncertain whether genomic instability precedes and causes the development of pulmonary arterial hypertension, which occurs through a process that can span three to five decades. Furthermore, it remains unclear whether the impairment of bone morphogenetic protein/BMPR2 signaling is involved in the susceptibility to genomic instability.

DNA double-strand breaks are considered highly damaging in many tissues, including endothelial cells, and require prompt and accurate repair^15^. Homologous recombination is the primary mechanism involved in DNA double-strand break repair^16,17^. RAD51 is an essential factor in DNA double-strand break repair, acting through gene conversion^18^ and participating in sister chromatin exchange in mammalian cells^18^. Upon genotoxic stress, RAD51 is recruited to DNA damage sites where it mediates the search for a homologous sequence during homologous recombination^19^. RAD51 also plays a critical role in stabilizing the DNA replication fork by promoting survival of replication stress and preventing accumulation of replication-associated DNA double-strand breaks^20^. Loss-of-function mutations or reduction of RAD51 lead to deregulation of homologous recombination, which results in increased sensitivity to DNA damaging agents and increased genetic rearrangements^21^, suggesting that cellular RAD51 is regulated to ensure proper execution of homologous recombination and the maintenance of genome integrity^22^. It has been reported that endothelial cells from pulmonary arterial hypertension patients and pulmonary microvascular endothelial cells with reduced BMPR2 protein are more sensitive to DNA damage due to decreased amounts of BRCA1 and DNA Topoisomerase II binding protein 1, both of which have critical roles in relaying the DNA damage signal^23,24^.

In this study, we show that the depletion or inhibition of BMPR2 activity leads to a decrease of RAD51 and an increase of DNA damage. Both can be rescued by stimulation with BMP9. We demonstrate that loss-of-function mutations in *BMPR2* result in genome instability and mutations, which contribute to the development of pulmonary vascular lesions through RAD51 deficiency.

## Results

### Loss of BMPR2 leads to depletion of RAD51

**Fig. 1:**
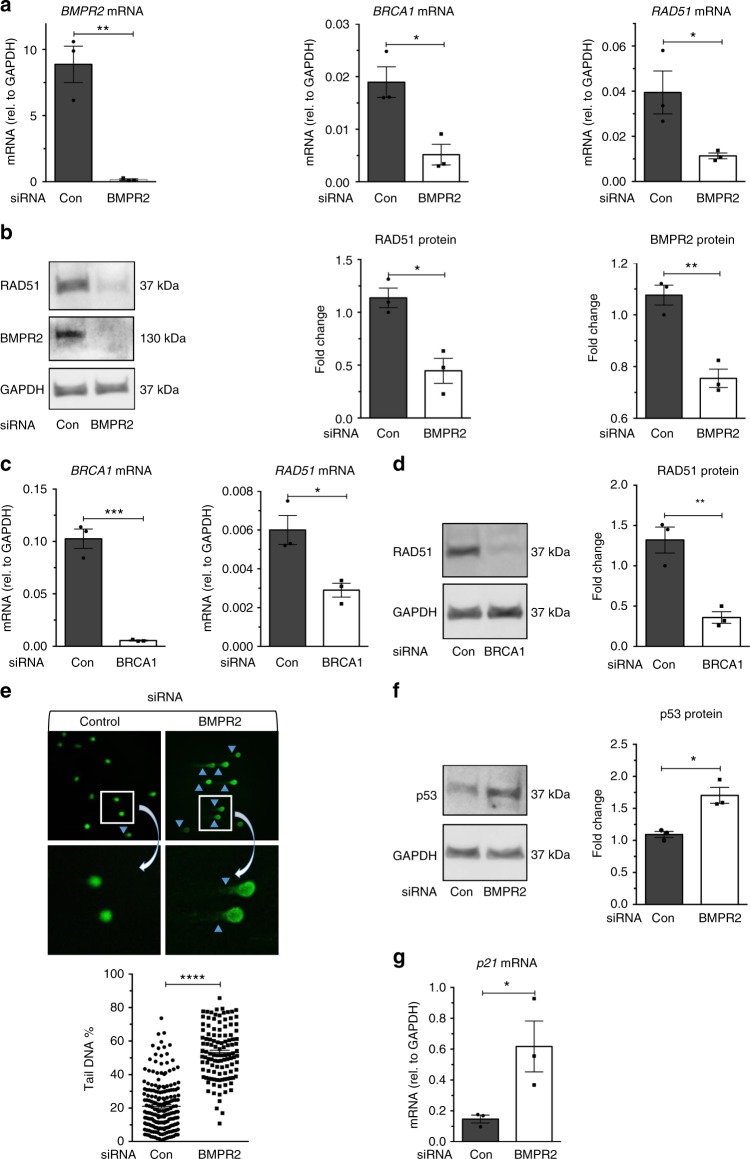
Ablation of BMPR2 results in a reduction of RAD51 and promotes DNA damage. **a** The amount of *BMPR2*, *BRCA1*, and *RAD51* mRNA relative to *GAPDH* mRNA was measured 48 h after control (Con) or *BMPR2* siRNA transfection initiations using quantitative PCR analysis (*n* = 3). **b** The amount of RAD51, BMPR2, and GAPDH (loading control) protein in Con or *BMPR2* siRNA-transfected pulmonary microvascular endothelial cells (PMVECs). GAPDH was used for normalization. Representative image and the quantitations of three independent experiments are shown (*n* = 3). **c** The level of *BRCA1* and *RAD51* mRNA relative to *GAPDH* mRNA in Con or BRCA1 siRNA-transfected PMVECs (*n* = 3). **d** RAD51 and GAPDH (loading control) protein amount in Con or *BRCA1* siRNA-transfected PMVECs were analyzed. Representative image and the quantitation of three independent experiments are shown (*n* = 3). **e** DNA damage of control (Con) or *BMPR2* siRNA-transfected PMVECs were analyzed 48 h after siRNA transfection initiation using alkaline single-cell gel electrophoresis (alkaline comet assay). The fraction (%) of cells with DNA damage (arrow heads) was analyzed by ImageJ software. Representative images of alkaline comet assay and the quantitation of 116–204 cells are shown. **f** p53 protein amount (*n* = 3) and **g** *p21* mRNA expression (*n* = 3) in Con or *BMPR2* siRNA-transfected PMVECs was examined. GAPDH was used for normalization. Bars represent mean ± SEM from three different experiments per conditions in (**a**–**g**). *P < 0.05, **P < 0.01, ***P < 0.001, and ****P < 0.0001 versus respective control. Unpaired two-tailed *t-*test was used in (**a**–**g**)

An attenuation of BRCA1 can be detected both in human primary pulmonary microvascular endothelial cells in which BMPR2 has been downregulated and in endothelium from idiopathic pulmonary arterial hypertension patients, indicating a link between deregulation of the BMPR2 signaling pathway and the level of BRCA1^23^. As BRCA1 is known to activate RAD51 and promote homologous recombination—a relatively error-free DNA damage response mechanism^16,25^—we hypothesized that decrease or loss of BMPR2 leads to deregulation of the homologous recombination repair mechanism through decreased RAD51. By transfecting small interfering RNA (siRNA) against *BMPR2* (*si-BMPR2*) into human pulmonary microvascular endothelial cells, we confirmed a 98% reduction of *BMPR2* messenger RNA (mRNA; *P* = 0.0032) and an ~70% reduction of BMPR2 protein (*P* = 0.0035) using quantitative PCR and immunoblotting (Fig. 1a, b). The reduced *BMPR2* amount correlated with a 72.8% reduction of *BRCA1* mRNA (*P* = 0.0172, Fig. 1a), consistent with previous results^23^. Given the established link between BRCA1 and RAD51^25^, we investigated the role and function of RAD51 in pulmonary microvascular endothelial cells upon downregulation of BMPR2. At 48 h after transfection of *si-BMPR2*, pulmonary microvascular endothelial cells showed a 71% reduction of *RAD51* mRNA (*P* = 0.042, Fig. 1a) and a 60.7% reduction of RAD51 protein (*P* = 0.0101, Fig. 1b) compared to pulmonary microvascular endothelial cells transfected with non-targeting control siRNA (Fig 1a, b). Similarly, when *si-BRCA1* was transfected into pulmonary microvascular endothelial cells and the level of *BRCA1* mRNA was reduced by 94.6% (*P* = 0.0005, Fig. 1c), *RAD51* mRNA and protein were reduced to 48% (*P* = 0.0204, Fig. 1c) and 73% (*P* = 0.0055, Fig. 1d), respectively, compared to control pulmonary microvascular endothelial cells. These results suggest that reduced BMPR2 in pulmonary microvascular endothelial cells can lead to decreased RAD51. Thus, we examined the extent of DNA damage as a result of the decrease in RAD51 using the alkaline single-cell gel electrophoresis assay (hereafter referred to as alkaline comet assay), which detects single-strand breaks, double-strand breaks, and alkaline-labile sites in DNA^26^. *Si-BMPR2*-transfected pulmonary microvascular endothelial cells showed a 61% higher level of fragmented DNA that could be electrophoresed away from nuclei (tail DNA), a sign of DNA damage, compared to control siRNA-transfected pulmonary microvascular endothelial cells (*P* < 0.0001, Fig. 1e). Upon DNA damage, the tumor suppressor protein TP53 (hereafter referred to as p53) is rapidly induced and mediates multiple responses, including DNA repair, cell cycle arrest, apoptosis, and senescence^27^. Furthermore, reduction of homologous recombination activity and of RAD51 has been shown to result in increased p53^28^. Thus, we hypothesized that reduction of BMPR2 could induce p53. As expected, *si-BMPR2*-transfected pulmonary microvascular endothelial cells showed an ~36% increase in p53 protein compared to control siRNA-transfected cells (*P* = 0.0010, Fig. 1f), which is similar to the increase observed in endothelial cells from the endothelial cell-specific *BMPR2* knockout mouse^29^. Consistent with the induction of p53, *p21* mRNA, a transcriptional target of p53, was induced fourfold in *si-BMPR2* cells compared to control (*P* = 0.047, Fig. 1g). These results demonstrate that loss of expression of BMPR2 by siRNA leads to the depletion of RAD51 and accumulation of DNA damage in pulmonary microvascular endothelial cells.

### DNA damaging agents lead to RAD51 deficiency

**Fig. 2:**
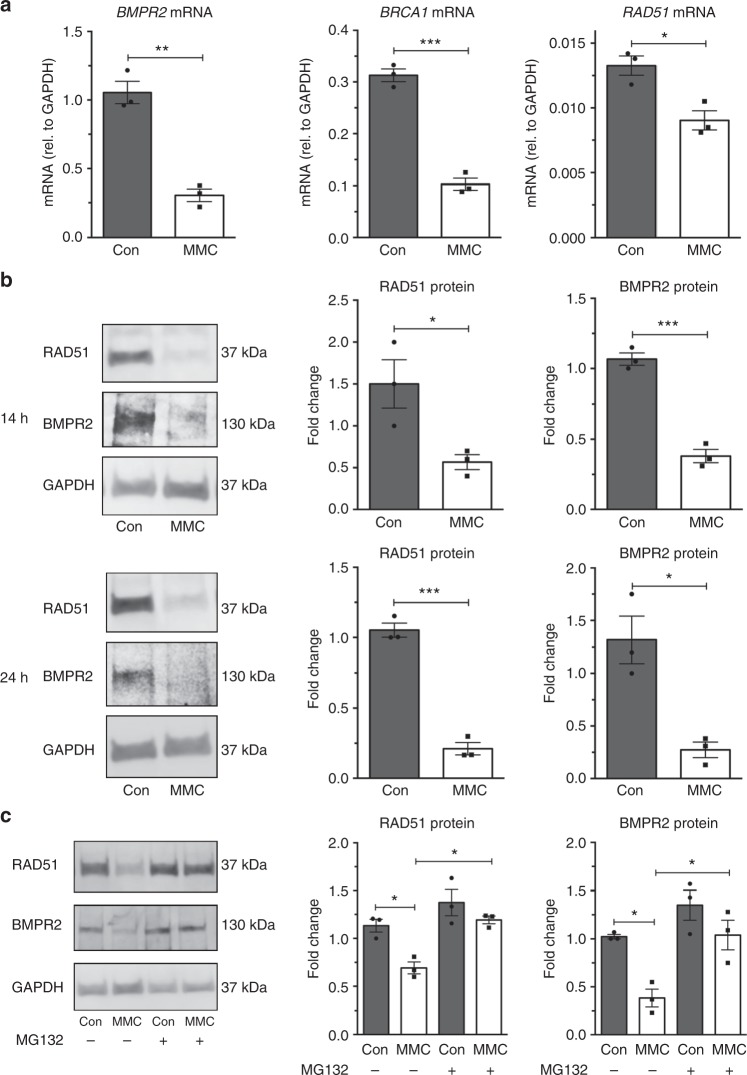
DNA damage leads to a depletion of BMPR2 and RAD51. **a** Pulmonary microvascular endothelial cells (PMVECs) were treated with DNA damaging agent mitomycin C (MMC) or vehicle (H_2_O, Con) for 14 h followed by qRT-PCR analysis of *BMPR2*, *BRCA1*, and *RAD51* mRNA relative to *GAPDH* mRNA (*n* = 3). **b** PMVECs were treated with MMC or vehicle (H_2_O, Con) for 14 h or 24 h, followed by immunoblot analysis of RAD51, BMPR2, and GAPDH (loading control). Representative image and the quantitation of three independent experiments are shown (*n* = 3). **c** RAD51, BMPR2, and GAPDH (loading control) protein amount in PMVECs treated with vehicle (H_2_O, Con) or MMC with or without proteasome inhibitor MG-132 were analyzed by immunoblot. Representative image and the quantitation of three independent experiments are shown (*n* = 3). Bars represent mean ± SEM from three different experiments per conditions in (**a**–**c**). *P < 0.05, **P < 0.01, and ***P < 0.001 versus respective control. Unpaired two-tail *t*-test was used in (**a**, **b**). One-way ANOVA followed by Tukey’s multiple comparisons test was used in (**c**)

We previously showed that the treatment of pulmonary microvascular endothelial cells with DNA damaging chemotherapy agents, such as mitomycin C, induces a rapid reduction of BMPR2 and BRCA1^23^. When pulmonary microvascular endothelial cells were treated with mitomycin C (50 µg/mL) for 14 h, *BMPR2*, *BRCA1*, and *RAD51* mRNAs were reduced to 71% (*P* = 0.013, Fig. 2a), 67% (*P* = 0.0002, Fig. 2a), and 38% (*P* *=* 0.0157, Fig. 2a), respectively, when compared to control cells. Concurrently, the amount of BMPR2 and RAD51 proteins was reduced by 64 and 62%, respectively (*P* = 0.0004 and *P* = 0.0365, Fig. 2b, 14 h). A similar reduction of BMPR2 and RAD51 proteins was observed after 24 h of mitomycin C treatment (*P* = 0.0075 and *P* = 0.0002, Fig. 2b, 24 h) and 6 h of camptothecin treatment (*P* = 0.0004 and *P* = 0.0331, Supplementary Fig. 1). Conversely, when pulmonary microvascular endothelial cells were treated with the proteasome inhibitor MG-132 to prevent the degradation of BMPR2 protein induced by mitomycin C (*P* *<* 0.05, Fig. 2c), RAD51 protein was increased 1.7-fold (*P* < 0.05, Fig. 2c). These results support the hypothesis that DNA damage agent-induced RAD51 deficiency in pulmonary microvascular endothelial cells might be caused indirectly by downregulation of BMPR2.

### RAD51 deficiency promotes DNA damage

**Fig. 3:**
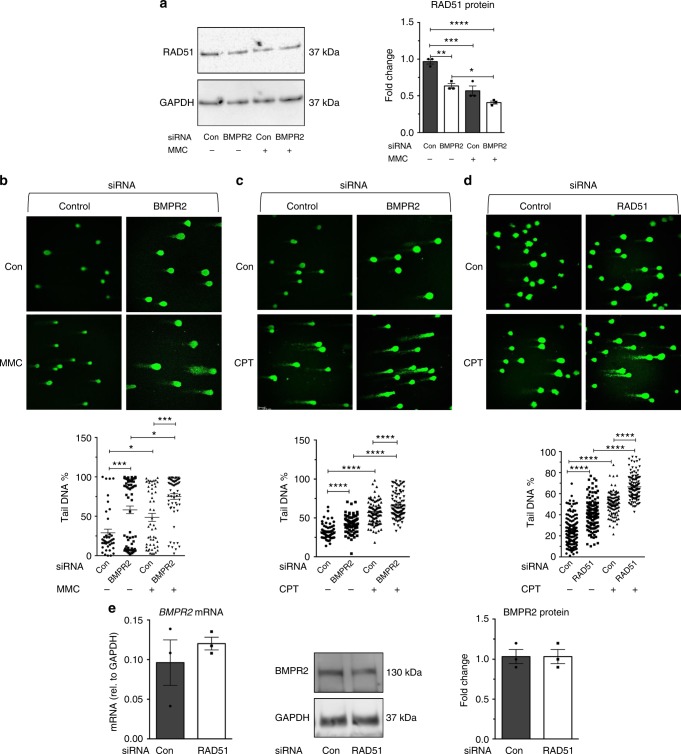
Maintenance of RAD51 and genome integrity is controlled by BMPR2 signaling pathway. Pulmonary microvascular endothelial cells (PMVECs) were transfected with Control (Con), *BMPR2*, or *RAD51* siRNAs. After 24 h, PMVECs were treated with mitomycin C (MMC) for 14 h or camptothecin (CPT) for 6 h. **a** The amount of RAD51 protein normalized to GAPDH was measured in PMVECs transfected with Con or *BMPR2* siRNAs and treated with MMC or vehicle (H_2_O, Con) for 14 h. Representative image and the quantitation of three independent experiments are shown (*n* = 3). **b** Single-cell gel electrophoresis (alkaline comet assay) was performed using Con or *BMPR2* siRNA-transfected PMVECs after vehicle (Con) or MMC treatment for 14 h and the fraction (%) of cells with DNA damage was quantitated by ImageJ software. Representative images of alkaline comet assay and the quantitation of 60–70 cells are shown. **c** Alkaline comet assay was performed using Con or *BMPR2* siRNA-transfected PMVECs after vehicle (DMSO, Con) or CPT treatment for 6 h and the degree of DNA damage in cells was examined. Representative images and the quantitation of 70–100 cells are shown. **d** Alkaline comet assay was performed using Con or *RAD51* siRNA-transfected PMVECs after vehicle (Con) or CPT treatment for 6 h and the degree of DNA damaged cells was compared (*n* = 100–160). **e** Con or *RAD51* siRNA was transfected in PMVECs, followed by quantitation of *BMPR2* mRNA and BMPR2 protein (*n* = 3). GAPDH was used for normalization. Bars represent mean ± SEM from three different experiments per conditions in (**a**–**e**). *P < 0.05, **P < 0.01, ***P < 0.001, and ****P < 0.0001 versus a respective control. One-way ANOVA followed by Tukey’s multiple comparisons test was used in (**a**–**d**). Unpaired two-tail *t*-test was used in (**e**)

Next, we performed alkaline comet assay to compare the sensitivity of pulmonary microvascular endothelial cells to DNA damage agents in the presence or absence of BMPR2. After mitomycin C treatment, the amount of RAD51 protein in *si-BMPR2*-treated cells was 28% lower than in control siRNA-treated cells (*P* < 0.05, Fig. 3a). We observed a concomitant 22.6% increase of DNA damage in *si-BMPR2* cells compared to control cells upon mitomycin C treatment (*P* < 0.001, Fig. 3b). A similar result was obtained following treatment with camptothecin, a different DNA damaging agent, upon which the extent of DNA damage measured by alkaline comet assay was 14% higher in *si-BMPR2* cells than in controls (*P* < 0.0001, Fig. 3c). These results further support the hypothesis that pulmonary microvascular endothelial cells with reduced BMPR2 are more susceptible to DNA damaging agents^23^. The level of DNA damage in *si-BMPR2* cells after camptothecin treatment was comparable to that borne by cells transfected with *si-RAD51* (23.6%, *P* < 0.0001, Fig. 3d). Upon downregulation of *RAD51* mRNA (*P* *=* 0.049, Supplementary Fig. 2a) and protein by siRNA (*P* *=* 0.0006, Supplementary Fig. 2b), *BMPR2* mRNA and protein (Fig. 3e) were unchanged, suggesting that BMPR2 is an upstream regulator of RAD51. These results support a causal relationship between RAD51 deficiency and accumulation of DNA damage in pulmonary microvascular endothelial cells.

### BMP9 treatment rescues RAD51 deficit and DNA damage

**Fig. 4:**
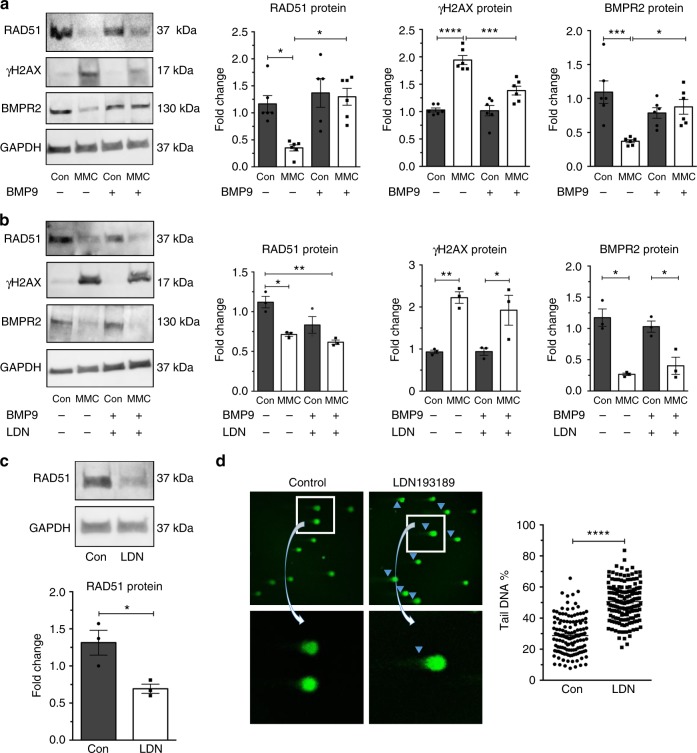
Activation of BMPR2 signaling by BMP9 partially rescues RAD51 and reduces sensitivity to DNA damage agent. **a** Pulmonary microvascular endothelial cells (PMVECs) were treated with vehicle (Con, H_2_O) or mitomycin C (MMC) with or without BMP9 (10 ng/mL) for 14 h. RAD51 and BMPR2 protein relative to GAPDH were examined by immunoblot. γH2AX was studied to measure the amount of double-strand breaks. Representative image and the quantitation of six independent experiments (*n* = 6) are shown. **b** RAD51 and BMPR2 protein amount in PMVECs treated with vehicle (H_2_O, Con) or MMC with or without bone morphogenetic protein receptor kinase inhibitor LDN193189 (LDN; 100 nM) in combination with BMP9 for 14 h were examined by immunoblot and the quantitation of three independent experiments are shown (*n* = 3). γH2AX represents the amount of double-strand breaks. GAPDH was used for normalization. **c** RAD51 and GAPDH (loading control) protein amount in PMVECs treated with or without LDN193189 (100 nM) for 72 h. Representative image and the quantitation of three independent experiments are shown (*n* = 3). **d** PMVECs were treated with or without LDN193189 (100 nM) for 72 h, and the amount of DNA damage was measured by single-cell gel electrophoresis (alkaline comet assay). The percentage of cells with DNA damage was analyzed using ImageJ software. Representative images of alkaline comet assay and the quantitation of 136–162 cells are shown. Bars represent mean ± SEM from six different experiments per conditions in (**a**) and from three different experiments per conditions in (**b**–**d**). *P < 0.05, **P < 0.01, ***P < 0.001, and ****P < 0.0001 versus respective control. One-way ANOVA followed by Tukey’s multiple comparisons test was used in (**a**, **b**). Unpaired two-tail *t*-test was used in (**c**, **d**)

A recent study reported that selective enhancement of endothelial BMPR2 signaling by BMP9 was able to rescue endothelial cells from apoptosis and pulmonary arterial hypertension in mice carrying a heterozygous *BMPR2* mutation^30^. When pulmonary microvascular endothelial cells were treated with BMP9, cells were protected from the mitomycin C-mediated decrease in *BMPR2* and *RAD51* mRNAs (*P* < 0.0001 and *P* < 0.001, Supplementary Fig. 3a) and proteins (*P* < 0.05, Fig. 4a). Induction by mitomycin C of γH2AX, a marker of DNA damage, was abolished by co-treatment with BMP9 (*P* < 0.001, Fig. 4a). Unlike BMP9 (Fig. 4a), BMP4 was unable to inhibit mitomycin C-mediated BMPR2 and RAD51 depletion and γH2AX induction (Supplementary Fig. 3b). Consistently, the relative amount of phospho-SMAD1/5/8 in pulmonary microvascular endothelial cells stimulated with 10 ng/mL BMP4 was ~42% lower than in cells stimulated with 10 ng/mL BMP9, indicating that BMP4 is less potent than BMP9 on endothelial cells (*P* < 0.05 and *P* < 0.001, Supplementary Fig. 3c). When pulmonary microvascular endothelial cells were treated with a small molecule inhibitor of bone morphogenetic protein signaling, LDN193189^31^, the depletion of BMPR2 and RAD51 by mitomycin C was no longer rescued by BMP9 stimulation (Fig. 4b). Concurrently, the mitomycin C-mediated induction of DNA damage (γH2AX) was no longer rescued by BMP9 (Fig. 4b). These results suggest that the BMP signaling pathway, inhibited by LDN193189, plays a role in the rescue of RAD51 and DNA damage by BMP9. Furthermore, when pulmonary microvascular endothelial cells were treated with a low concentration of LDN193189 for 72 h, which inhibited the BMP9–BMPR2–SMAD signaling axis based on the suppression of the bone morphogenetic protein targets *ID1* and *ID3 mRNAs* (*P* = 0.0079 and *P* = 0.048, Supplementary Fig. 4)^32^, RAD51 protein was reduced by ~47% (*P* = 0.0253, Fig. 4c), and DNA damage was increased by 43%, as measured by alkaline comet assay (*P* < 0.0001, Fig. 4d). Similarly, both the chemical inhibition of the endothelial-specific type I BMP receptor ALK-1 (also known as ACVRL1) by K02288^33^, confirmed by ~40% reduction of SMAD1/5/8 phosphorylation (*P* = 0.0006, Supplementary Fig. 5a), and the knockdown of *ALK-1* by siRNA (*P* = 0.0004, Supplementary Fig. 5b) in pulmonary microvascular endothelial cells resulted in a reduction of RAD51 protein by ~31% (*P* *=* 0.0056, Supplementary Fig. 5a) and 42% (*P* *=* 0.0376, Supplementary Fig. 5b), respectively. Finally, the depletion of the signal transducers SMAD1 and SMAD5 (*P* *=* 0.0005 and *P* *=* 0.0084, Supplementary Fig. 5c) led to a small reduction of RAD51 (Supplementary Fig. 5c). Altogether, these results support a critical role of the BMP9–ALK-1–BMPR2 signaling axis in the maintenance of RAD51 and the protection of genome integrity in pulmonary microvascular endothelial cells.

### Downregulation of the *microRNA-96*–RAD51 axis by BMP signaling

**Fig. 5:**
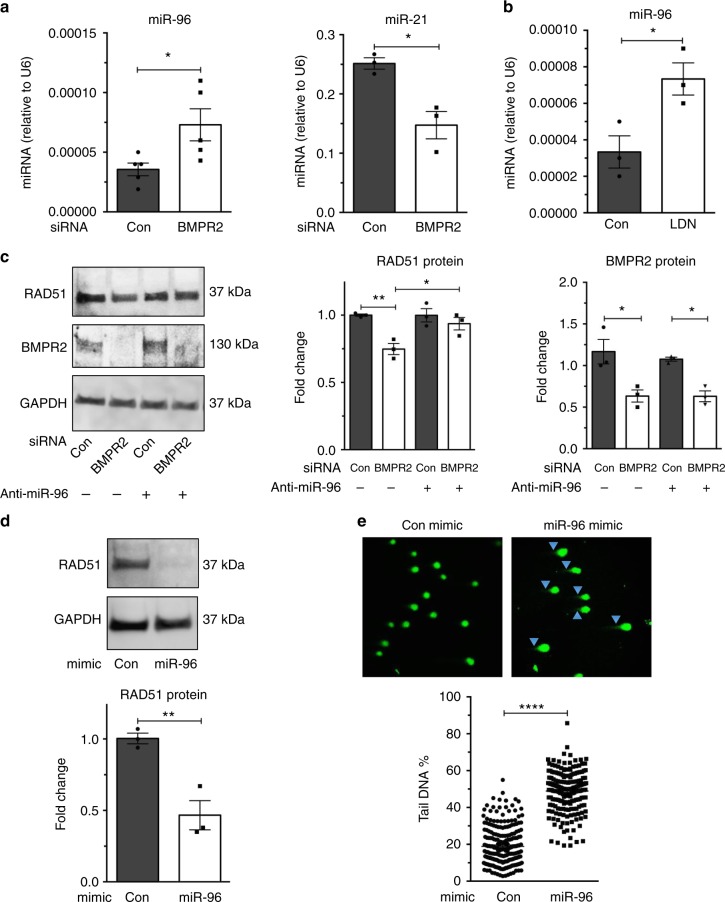
Increased *miR-96* upon depletion of BMPR2 promotes attenuation of RAD51. **a** Pulmonary microvascular endothelial cells (PMVECs) were transfected with control (Con) or BMPR2 siRNAs for 48 h, followed by quantitation of *miR-96* (*n* = 5) and *miR-21* (*n* = 3) relative to *U6 snRNA* measured by quantitative PCR analysis. **b** PMVECs were treated with vehicle (DMSO, Con) or LDN193189 (LDN; 100 nM) for 72 h followed by quantitation of *miR-96* relative to U6 snRNA (*n* = 3). **c** RAD51 and BMPR2 protein in PMVECs co-transfected with Con or BMPR2 siRNAs with anti-*miR-96* or control anti-miRNA. **d** PMVECs were transfected with control mimic (Con) or *miR-96* mimic (miR-96) for 48 h, followed by quantitation of RAD51 by immunoblot (*n* = 3) and **e** the amount of DNA damage was measured using alkaline comet assay from 204–235 cells using ImageJ software. Bars represent mean ± SEM from five different experiments per conditions in (**a**) (miR-96) and from three different experiments per conditions in (**a**) (miR-21) and (**b**–**e**). *P < 0.05, **P < 0.01, and ****P < 0.001 versus respective control. Unpaired two-tailed *t*-test was used in (**a**, **b**) and (**d**–**e**). One-way ANOVA followed by Tukey’s multiple comparisons test was used in (**c**)

We previously showed that a small noncoding *microRNA-96* (*miR-96*) expression is repressed upon stimulation of the bone morphogenetic protein signaling pathway in pulmonary artery smooth muscle cells^34^. *miR-96* targets the *RAD51* mRNA and reduces its expression^35^. We hypothesized that the BMP9–ALK-1/BMPR2–SMAD axis is responsible for the decrease of *miR-96* also in pulmonary microvascular endothelial cells, thus allowing RAD51 to be expressed. When BMPR2 was inhibited by si-BMPR2, the amount of *miR-96* was increased 2-fold (*P* *=* 0.0322, Fig. 5a), as also measured in pulmonary artery smooth muscle cells^34^. *miR-21*, whose expression is induced by the BMP-SMAD pathway^36^, was decreased by 41.4% upon inhibition of BMPR2 by si-BMPR2 (*P* *=* 0.0140, Fig. 5a). When pulmonary microvascular endothelial cells were treated with LDN193189 and the bone morphogenetic protein signaling pathway was attenuated (*P* *=* 0.0079 and *P* *=* 0.048, Supplementary Fig. 4), *miR-96* level increased by 54% (*P* = 0.0327, Fig. 5b). Similarly, when *ALK-1* was reduced by siRNA (*P* *=* 0.004, Supplementary Fig. 6a), *miR-96* increased by ~23% (*P* *=* 0.0488, Supplementary Fig. 6a). Inactivation of *miR-96* by transfection of antisense oligonucleotides (anti-miR-96, *P* *=* 0.01, Supplementary Fig. 6b) prevented the reduction of *RAD51* mRNA (*P* *<* 0.01, Supplementary Fig. 6c) and protein (*P* *<* 0.05, Fig. 5c) by *si-BMPR2* (*P* *<* 0.01 Supplementary Fig. 6c and *P* *<* 0.05, Fig. 5c). Conversely, exogenous expression of *miR-96* (*P* *=* 0.05, Supplementary Fig. 6d) led to ~54% reduction of RAD51 protein (*P* = 0.0078, Fig. 5d) and increased the amount of DNA damage in pulmonary microvascular endothelial cells (*P* *=* 0.0001, Fig. 5e). These results suggest that constitutive suppression of *miR-96* by the BMP9–BMPR2 signaling axis is essential to support the expression of RAD51 and maintain a sufficient level of DNA repair in pulmonary microvascular endothelial cells.

### Reduction of RAD51 in animal models of pulmonary arterial hypertension and human patients

**Fig. 6:**
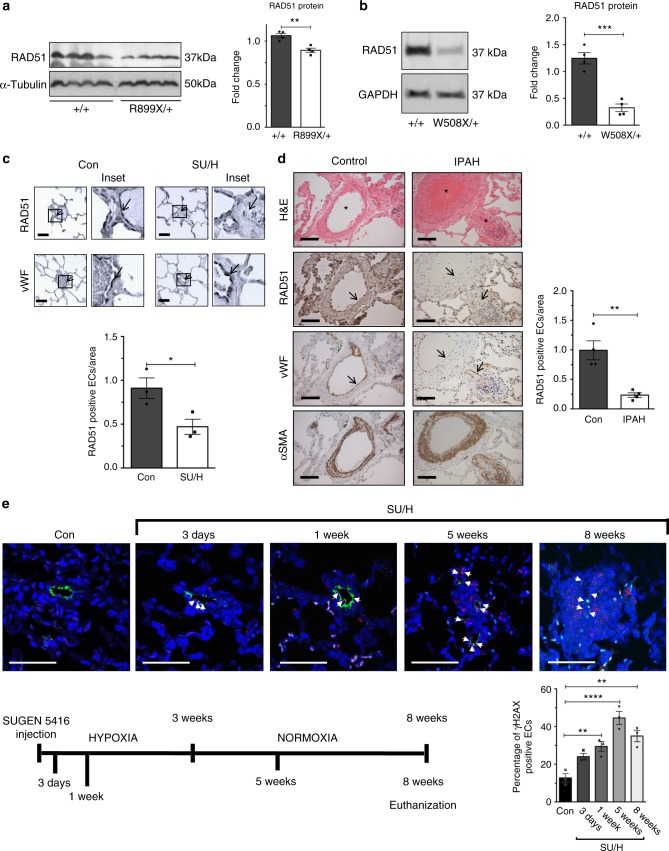
Attenuation of RAD51 in animal models and human patients of pulmonary arterial hypertension. **a** RAD51 and α-tubulin (loading control) protein amount was measured in whole lung lysates from 6-month-old *BMPR2 R899X* mutant mice (*R899X*/+) or littermate controls (+/+). Representative image of the blot and the quantitation of four individual samples are shown (*n* = 4). **b** RAD51 protein amount in pulmonary arterial smooth muscle cells isolated from control rats (+/+) or rats with heterozygous *BMPR2 W508X* mutation (*W508X*/+) were examined. Representative image of immunoblot and the quantitation of four independent experiments are shown (*n* = 4). **c** Representative images of immunohistochemistry of RAD51 and von Willebrand factor (vWF), a marker for endothelial cells, in lungs isolated from male control and SUGEN-5416/chronic hypoxia (SU/H)-treated rats (*n* = 3). Arrows indicate the endothelium of pulmonary arteries. Scale bars indicate 25 μm. **d** Representative images of hematoxylin and eosin (H&E) and immunohistochemical staining for RAD51 (200×), vWF, and α-smooth muscle actin (αSMA), a marker for smooth muscle cells, in lungs isolated from a control individual and an idiopathic pulmonary arterial hypertension patient (IPAH) (*n* = 4). Asterisks and arrows indicate the location of pulmonary arteries, pulmonary endothelium, and pulmonary artery smooth muscle cells, respectively. Scale bars indicate 100 μm. **e** Representative images of immunofluorescence staining of DNA damage marker γH2AX (red), endothelial marker, vWF (green), and DAPI in lungs from control (Con) and SU/H-treated rats (*n* = 3). Schematic diagram of SU/H treatment and different time points of sampling lungs are indicated. The relative amount of γH2AX signal in the endothelium was quantitated. Scale bar indicate 50 μm. Bars represent mean ± SEM from four different experiments per conditions in (**a**, **b**), and (**d**), and from three different experiments per conditions in (**c**) and (**e**). *P < 0.05, **P < 0.01, ***P < 0.001 and ****P < 0.0001 versus respective control. Unpaired two-tail *t*-test was used in (**a**–**d**). One-way ANOVA followed by Tukey’s multiple comparisons test was used in (**e**)

To investigate in vivo the status of RAD51 in the presence of a loss-of-function mutation in *BMPR2*, we measured the RAD51 protein by immunoblotting using whole lung lysates from wild-type mice (+/+) or from mutant mice with reduced BMPR2 expression due to a mutation of arginine 899 into a stop codon in one allele of *BMPR2* (*R899X/*+)^30^. Around 6 months of age, *R899X*/+ mice develop mild pulmonary arterial hypertension and mimic the genetic background observed in human pulmonary arterial hypertension patients^30^. Interestingly, 6-month-old *R899X*/+ mice showed a 16% reduction of RAD51 protein compared to control littermates (+/+) (*P* = 0.0033, Fig. 6a). To address the species- and cell-type specificity of this effect, we evaluated whether loss of function of *BMPR2* also results in a decrease of RAD51 in rat pulmonary artery smooth muscle cells. Indeed, the reduction of RAD51 was not specific for pulmonary microvascular endothelial cells, as pulmonary artery smooth muscle cells isolated from rats with the heterozygous *BMPR2* mutation W508X (*W508X*/+), producing an early stop codon and an ~47% reduction of BMPR2 protein (*P* *=* 0.0022, Supplementary Fig. 7a)^37^, showed a 68% decrease in *RAD51* mRNA *(P* = 0.0395, Supplementary Fig. 7b) and a 74% decrease in RAD51 protein (*P* < 0.0004, Fig. 6b) compared to control rats (+/+). This result suggests that the regulatory link between BMPR2 and RAD51 can be observed in vivo in different vascular cell types and rodent species. As *R899X*/+ mice carry the heterozygous *BMPR2* mutation in all tissues, we further examined whether loss of function of BMPR2 mediates DNA damage in tissues other than pulmonary vascular cells, such as the liver and the right ventricle. Interestingly, although we detected an ~2.2-fold (*P* = 0.0051, Supplementary Fig. 8a) and an ~1.8-fold (*P* = 0.0814, Supplementary Fig. 8b) increase in DNA damage in the liver and the right ventricle of *R899X*/+ mice, respectively, we did not find a significant change in the amount of RAD51 protein (Supplementary Fig. 8a and 8b). This result suggests that RAD51 may play a more critical role in the protection of genome integrity in response to BMPR2 signaling in pulmonary vascular cells, while other factors may mediate the effect of BMPR2 on DNA damage in liver and heart cells.

**Fig. 7:**
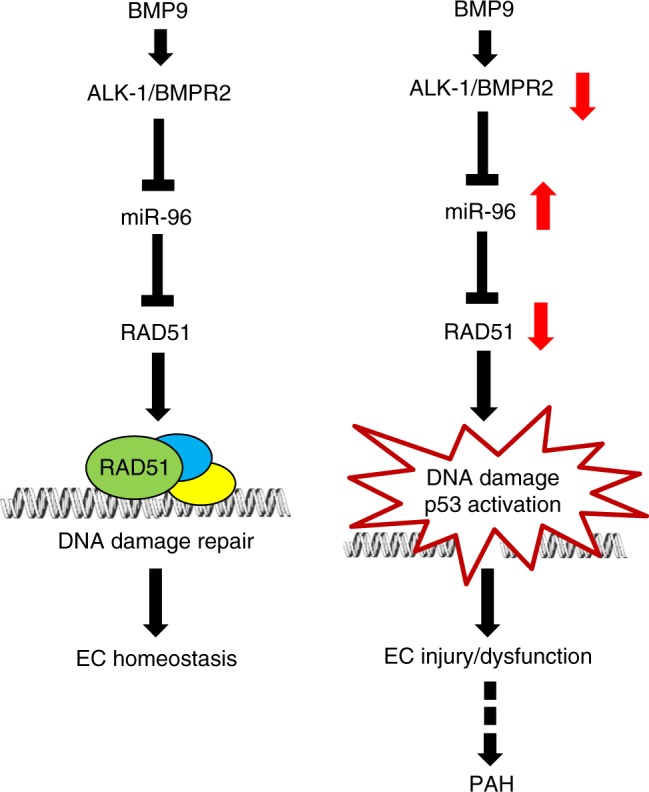
Schematic diagram of the maintenance of RAD51 by the BMP9–ALK-1/BMPR2 signaling axis. In healthy individuals, RAD51 is maintained in the pulmonary endothelium as the BMP9–ALK-1/BMPR2 signaling axis downregulates *miR-96* expression and prevents the destabilization of *RAD51* mRNA. When the BMP9–ALK-1/BMPR2 signaling axis is deregulated, elevation of *miR-96* leads to attenuation of RAD51. As a result, DNA damage accumulates in the pulmonary endothelium over several decades causing endothelial injury and dysfunction, and eventually triggers pulmonary vascular remodeling

Finally, we examined whether attenuation of RAD51 could be observed in a non-genetic model of pulmonary arterial hypertension, the Sugen–Hypoxia^38^-treated rats, and in human patients affected by idiopathic pulmonary arterial hypertension. The Sugen–Hypoxia treatment consists of exposure to a single dose of SUGEN-5416, followed by 3 weeks in hypoxia (10% O_2_) and additional 5 weeks in normoxia (total of 8 weeks)^39^. It has been reported that Sugen–Hypoxia-treated rats exhibit both a depletion of BMPR2 and elevated DNA damage^39,40^. Immunohistochemical analysis of RAD51 in the lung sections of Sugen–Hypoxia-treated rats compared to control rats showed 50% reduction (*P* *<* 0.0390) of RAD51 in the endothelium, identified by the endothelial marker von Willebrand Factor (Fig. 6c). Similarly, immunohistochemistry of lung sections from idiopathic pulmonary arterial hypertension patients showed 76% reduction of RAD51 (*P* *=* 0.0038) in the endothelium of pulmonary arteries compared to control samples (Fig. 6d). To address the kinetics of RAD51 regulation, we measured the induction of DNA damage in the pulmonary endothelium in the Sugen–Hypoxia-treated rat model at various time points and found that the increase in DNA damage was detected as early as 3 days (Fig. 6e) and significantly increased after 1 week (*P* < 0.01, Fig. 6e) after Sugen–Hypoxia treatment, suggesting that DNA damage in the endothelium proceeds vascular remodeling, which can be detected by 5 weeks (3 weeks in hypoxia plus 2 weeks in normoxia) (Fig. 6e). The amount of DNA damage was further increased 5 weeks after the treatment (*P* < 0.0001, Fig. 6e), and remained stable until 8 weeks (3 weeks in hypoxia plus 5 weeks in normoxia), when it could also be detected in other vascular cells (Fig. 6e). These results suggest that DNA damage correlates with attenuation of BMPR2 and RAD51 in endothelial cells in rodents and humans, and it precedes pulmonary vascular remodeling in induced pulmonary arterial hypertension. At a later stage, the accumulation of DNA damage in both endothelial cells and other cells may aggravate the vascular lesions in pulmonary arterial hypertension (Fig. 7).

## Discussion

In this study, we uncovered a role of the bone morphogenetic protein signaling pathway in maintenance of genome stability through RAD51 protein, which prevents pathological remodeling. Bone morphogenetic protein signaling is essential for the regulation of vascular morphogenesis and function. Mutations that cause deregulation of bone morphogenetic protein signaling have been linked to at least two human vascular diseases, pulmonary arterial hypertension and hereditary hemorrhagic telangiectasia^41–43^. In particular, bone morphogenetic proteins are required for the differentiation and the structural and functional integrity of normal vascular endothelial cells^41–43^. Bone morphogenetic proteins exhibit pleiotropic activities through their downstream effectors, the SMAD proteins, which regulate gene expression by modulating transcription of both protein coding genes and noncoding microRNAs (miRNAs)^44,45^. Here we show that transcriptional suppression of *miR-96* by the bone morphogenetic protein pathway is critical for the expression of RAD51 and the prevention of DNA damage in pulmonary microvascular endothelial cells (Fig. 7), which in turn are fundamental to the integrity of the pulmonary vasculature.

It has been reported previously that female *R899X*/+ mice (6 months old) express less *miR-96* in pulmonary artery smooth muscle cells than control (+/+) female mice^46^. Consequently, the increased 5-hydroxytriptamine 1B receptor and serotonin mediate proliferation of pulmonary artery smooth muscle cells and promote pulmonary vascular remodeling in female *R899X*/+ mice^46^. Unlike female *R899X*/+ mice, male *R899X*/+ mice express the same amount of *miR-96* as +/+ mice, for unknown reasons^46^. It is unclear why *miR-96* is decreased in pulmonary artery smooth muscle cells from *R899X*/+ female mice, while knockdown of *BMPR2* or inhibition of bone morphogenetic protein signaling in pulmonary microvascular endothelial cells increases *miR-96*. We speculate that the discrepancy is, in part, due to a cell-type-specific response of pulmonary artery smooth muscle cells vs microvascular endothelial cells. It is also plausible that the genetic perturbation of the bone morphogenetic protein pathway in mice triggers a compensatory gene regulatory network that adapts to *BMPR2* haploinsufficiency, while the acute perturbation of the bone morphogenetic protein pathway would not allow sufficient time for genetic compensation^47^.

The inhibition of *miR-96* has also been shown to be critical in the context of the pathogenesis of Alzheimer’s disease, according to a study performed in brain microvascular endothelial cells in response to granulocyte-macrophage colony-stimulating factor^48^. Furthermore, *miR-96*-mediated downregulation of *RAD51* in cancer cell lines increases their sensitivity to chemotherapy agents and leads to cell death^35^, a result similar to our observations in pulmonary microvascular endothelial cells upon inhibition of bone morphogenetic protein signaling. Thus, we speculate that the suppression of *miR-96* to boost RAD51 expression by the bone morphogenetic protein–SMAD pathway may operate not only in pulmonary microvascular endothelial cells, but also in other cell types, whereby bone morphogenetic protein signaling would play a prevalent role as a guardian of genome integrity. Furthermore, *miR-96* expression in peripheral blood mononuclear cells has been reported to increase upon aging^49^, suggesting that *BMPR2* mutation carriers may experience a further age-dependent reduction of RAD51, which may correlate with pulmonary arterial hypertension progress with age. Finally, *miR-96* has been classified as an oncogenic miRNA because its overexpression has been found in various tumors^50^. It is unknown whether *miR-96* expression in tumors correlates with the extent of DNA damage. In addition to *miR-96*-*RAD51*-dependent regulation, it has been reported that the *miR-223*–*poly (ADP-ribose) polymerase 1* axis also plays an important role in DNA damage control^51^. In pulmonary artery smooth muscle cells from human and animal models of pulmonary arterial hypertension, *miR-223* is reduced, which promotes activation of poly (ADP-ribose) polymerase 1 and DNA damage, downregulation of *miR-204*, and the subsequent activation of transcription factors (nuclear factor of activated T cells and hypoxia-inducible factor 1-α), leading to aberrant proliferation of pulmonary artery smooth muscle cells^40,52^. It is plausible that the therapeutic delivery of exogenous *miR-223* and/or *miR-204* might ameliorate or prevent the development of vascular remodeling in pulmonary arterial hypertension, although efficient conveyance of miRNAs to pulmonary artery smooth muscle cells might be more challenging than to vascular endothelial cells.

Dysfunctions of BMPR2 have been associated with a hypoxic microenvironment^53,54^ and a metabolic switch from mitochondrial glucose oxidation to cytoplasmic glycolysis^55^. This metabolic change is followed by increased production of intracellular reactive oxygen species, such as superoxide and hydrogen peroxide, resulting in formation of oxidative DNA damage^23,55,56^. Under physiological conditions, levels of reactive oxygen species are kept in balance to prevent their harmful effect to the cells^57^. However, sustained increase of intracellular reactive oxygen species can result in single-strand breaks that are readily converted to double-strand breaks^15^. On the other hand, increased levels of intracellular reactive oxygen species can also indicate a response to DNA damage^58^. Both the exogenous DNA damage by DNA damaging agents and the endogenous DNA damage by increased production of reactive oxygen species could compromise the cellular DNA repair mechanisms^15^. Therefore, the maintenance of an effective DNA repair system is critical to prevent gene mutations and to further the maintenance of genomic stability^15^. An accumulation of mutations and genomic instability occur when specific cell cycle checkpoint pathways or DNA damage response components, such as RAD51, are deregulated, or when the damage-load overcomes the ability of the cells to restore the damage^15^. Interestingly, both pulmonary artery smooth muscle cells and endothelial cells in which BMPR2 is depleted show smaller mitochondria and are less variable in size than healthy cells^29,59^. Similarly, the downregulation of RAD51 is associated with a hypoxic environment^60^ and RAD51-depleted cells show a decreased size and a smaller number of mitochondria^61^, which leads to mitochondrial dysfunctions, such as decreased mitochondrial membrane potential and adenosine triphosphate production, and increased DNA degradation^61^. It has been previously reported that the reduction of BMPR2 in endothelial cells promotes abnormal mitochondria function and mitochondrial DNA damage, which results in endothelial cell apoptosis^29^. The deregulation of DNA damage response mechanism through the BRCA1–RAD51 axis by reduced BMPR2, followed by an increased amount of DNA damage, could at least in part explain the formation of apoptosis-resistant and hyper-proliferative endothelial cells. Damage to mitochondrial DNA might also need to be considered, as suggested by the recent finding that the accumulation of HSP90 in the mitochondria of pulmonary artery smooth muscle cells in animal models of pulmonary arterial hypertension is implicated in the maintenance of mitochondrial DNA integrity^62^. Although the status of mitochondrial DNA damage in pulmonary arterial hypertension patients with or without *BMPR2* mutations is currently unknown, it is plausible to speculate that the BMPR2–RAD51 axis may play a role in the maintenance of pulmonary vascular homeostasis by protecting the integrity of not only nuclear DNA but also mitochondrial DNA, since RAD51 functions both in the nucleus and in the mitochondria^63^.

In a recently published study, human distal pulmonary arteries and pulmonary artery smooth muscle cells isolated from pulmonary arterial hypertension patients were shown to have increased amounts of TP53 binding protein 1 and poly (ADP-ribose) polymerase 1^40^, which are associated with non-homologous end-joining^64^. In contrast to homologous recombination, non-homologous end-joining rejoins the DNA ends without DNA template, frequently leaving insertions and deletions at the breakpoint^15,65^. Furthermore, increased p53 is associated with decreased homologous recombination activity and reduced RAD51 expression and increased non-homologous end-joining^28,64^. These observations are consistent with our results, as reduced BMPR2 results in an increase of p53 both in culture and in animal models of pulmonary arterial hypertension^29^, and in reduced expression of RAD51. Taken together, these results suggest that endothelial cell dysfunction and cell death can be initiated by deregulation of homologous recombination through decreased RAD51 and BRCA1 and a switch to non-homologous end-joining, an error-prone DNA repair mechanism, leading to accumulation of DNA errors, mutations, rearrangements, and even chromosomal translocations, as seen in endothelial cells from plexiform lesions isolated from pulmonary arterial hypertension patients^13,14,66–68^. Increased DNA damage is not specific to pulmonary vasculature, as lymphoblastoid cells and peripheral blood cells derived from pulmonary arterial hypertension patients reportedly exhibit increased DNA damage and increased sensitivity to the chemotherapy agents etoposide and bleomycin^66^.

Both in vitro and in vivo studies have elucidated several molecular mechanisms by which bone morphogenetic protein signaling affects vascular endothelial cell homeostasis and its failure leads to the pathologies of pulmonary arterial hypertension and hereditary hemorrhagic telangiectasia^41,43^. Still, it remains unclear why deregulation of bone morphogenetic protein signaling would cause vascular remodeling only 30–40 years after birth, and not earlier. Here we propose that a potential long-term effect of the decrease of bone morphogenetic protein signaling is the accumulation of DNA damage and mutations due to RAD51 depletion mediated by *miR-96*. Reduction of RAD51 is observed not only in pulmonary microvascular endothelial cells but also in pulmonary artery smooth muscle cells isolated from rats with heterozygous *BMPR2* mutation and from human idiopathic pulmonary arterial hypertension patients. The cellular responses triggered by the downregulation of DNA repair, however, are cell-type specific. Pulmonary artery smooth muscle cells isolated from pulmonary arterial hypertension patients exhibit an increased amount and activation of poly (ADP-ribose) polymerase 1, which promotes the survival and proliferation of pulmonary artery smooth muscle cells^40^, while endothelial cells undergo apoptosis and endothelial cell dysfunction, as observed in pulmonary arterial hypertension patients^69^. In both monocrotaline and Sugen–Hypoxia-treated rat pulmonary arterial hypertension models, a 2-week treatment with the poly (ADP-ribose) polymerase 1 inhibitor ABT-888 after the establishment of the disease was able to reverse the pulmonary arterial hypertension phenotype^40^. These preclinical studies have led to a phase I clinical trial of a clinically approved poly (ADP-ribose) polymerase 1 inhibitor (olaparib) for the treatment of pulmonary arterial hypertension, which will be completed in 2019 (https://clinicaltrials.gov/ct2/show/NCT03251872).

BMP9, but not BMP4, is able to rescue the mitomycin C-mediated DNA damage in pulmonary microvascular endothelial cells. BMP9 is a potent ligand in endothelial cells, as it binds with high affinity to the type I bone morphogenetic protein receptor ALK-1, which is enriched in endothelial cells, in combination with BMPR2^41^. On the contrary, BMP4 preferentially binds bone morphogenetic protein receptor type 1A and bone morphogenetic protein receptor type 1B (also known as ALK-3 and ALK-6). The significance of the BMP9–ALK-1/BMPR2-mediated signal in endothelial cell is supported by the causal mutations identified in pulmonary arterial hypertension and hereditary hemorrhagic telangiectasia patients in the *ACVRL1* and *GDF2* genes, which encode ALK-1 and BMP9, respectively^70,71^. Although rare, mutations in the *ACVRL1* gene were identified in a subset of pulmonary arterial hypertension patients who also develop hereditary hemorrhagic telangiectasia^72,73^. Daily injections of BMP9 into a hereditary pulmonary arterial hypertension model mouse harboring a heterozygous R899X mutation in *BMPR2* ameliorates the established pulmonary arterial hypertension phenotype, supporting a therapeutic potential of BMP9 for pulmonary arterial hypertension^30^. Our findings pointing to a molecular link between BMP9 signaling and RAD51 open a new therapeutic avenue aimed at rescuing the DNA repair enzyme and preventing DNA damage as a way to halt or delay the development of vascular remodeling in pulmonary arterial hypertension from an early stage.

## Methods

### Cell culture

Commercially available human primary pulmonary microvascular endothelial cells were purchased from ScienCell Research Laboratories (#3000, Carlsbad, CA) and grown in gelatin-coated dishes on commercial EGM-2 media (Lonza Clonetics, Fisher Scientific) containing 5% fetal bovine serum (FBS) and growth factors. Pulmonary microvascular endothelial cells were used between passages 3 and 8. When noted, endothelial cells were treated with mitomycin C (50 μg/mL; 14 h) or camptothesin (4 μM; 6 h) (both purchased from Sigma-Aldrich, St. Louis, MO) to induce DNA damage. H_2_O or dimethy sulfoxide (DMSO) was used as a control. To induce BMP signaling, endothelial cells were treated with BMP9 (10 ng/mL) or BMP4 (10 ng/mL) (R&D Systems, Minneapolis, MN), or with vehicle for 14 h. To inhibit bone morphogenetic protein signaling, endothelial cells were treated with LDN193189 (100 nM) or K02288 (1 μM) for 72 h (Cayman Chemical, Ann Arbor, MI). DMSO was used as a control. To prevent protein degradation, MG-132 (100 nM) (Sigma-Aldrich) was used. Rat pulmonary smooth muscle cells (*BMPR2*/+(+/+) and *BMPR2+/W508X (W508X*/+) PAC1 cells (American Type Culture Correction, Manassas, VA) were grown on high glucose Dulbecco's modified Eagle's medium (HyClone^TM^, GE Healthcare Life Sciences) with 10% FBS and 1% penicillin–streptomycin. The creation of *W508X*/+ PAC1 cells has been previously described^37^. In brief, *BMPR2* mutation (*W508X*) was targeted on PAC1 cell line using transcription activator-like (TAL) effector nucleases (TALEN)-mediated targeted gene mutagenesis. The TAL Effector Nucleotide Targeter 2.0 (Cornell University, Ithaca, NY) was used to design TALEN arms. TALEN effectors were transfected to cells together with pcCDN3.1-eGFP for 48 h, after which eGFP-positive PAC1 cells were sorted using flow cytometry. After sorting, single cells were seeded into 96-well plate. Mutations present in each single-cell clone were examined by Sanger sequencing^37^. All cell lines used in the study were characterized with cell-specific antibodies and tested negative for mycoplasma, bacteria, yeast, and fungi.

### siRNA transfection

For siRNA transfections, pulmonary microvascular endothelial cells were divided 24 h before transfections. siRNA transfections were done as previously described^23^. In brief, a 9.3 μL Lipofectamine RNAiMAX was mixed in 750 μL of Opti-Minimum Essential Medium (Opti-MEM; Gibco, Thermo Scientific, Waltham, MA) and incubated for 5 min at room temperature. Non-targeting control, *BMPR2*, *BRCA1*, *RAD51*, *ACVRL1 (ALK-1*), *SMAD1*, or *SMAD5* siRNAs (all purchased from Dharmacon, Thermo Scientific) were mixed with 750 μL of Opti-MEM. The final concentration of used siRNA mixture was 100 nM. These two mixtures were then pooled and incubated for 20 min at room temperature. The mixture was added to endothelial cells and incubated at 37 °C for 5 h, after which 5 mL of EGM-2 media containing growth factors was added to the cells. siRNA-transfected endothelial cells were harvested at 48–72 h after the beginning of transfections. Knockdown of each gene was determined using quantitative reverse transcriptase PCR (qRT-PCR) and/or western immunoblotting. For overexpression of *miR-96*, pulmonary microvascular endothelial cells were transfected with control or miR-96 mimics with methods similar to those described for siRNA experiments. For *miR-96* inhibition, pulmonary microvascular endothelial cells were co-transfected with control or *BMPR2* siRNAs together with control anti-miR or anti-*miR-96*, with miRNA final concentration of 30 nM. The overexpression and inhibition of *miR-96* was determined using Taqman® microRNA Assay kit for *miR-96* (Thermo Fisher Scientific), as instructed by the manufacturer.

### Gene expression analysis

RNA isolation was done using the Qiagen RNeasy® Mini Kit (Qiagen GmbH, Hilden, Germany) and further reverse transcribed using iScript cDNA Synthesis Kit (BioRad Laboratories Inc., Hercules, CA) as instructed by the manufacturer. Quantitative PCR analysis was performed using PerfeCTa® SYBR Green FastMix® (Quantabio, Beverly, MA) with a BioRad CFX Connect^TM^ Real-Time system (BioRad Laboratories Inc.). Primer sets were purchased from Integrated DNA Technologies (IDT, Coralville, IA). Studied mRNA expression levels were normalized for *GAPDH* mRNA expression. For *miR-21* and *miR-96* expression analysis, the Taqman® microRNA Assay kit (Thermo Fisher Scientific) was used following the protocol provided by the manufacturer. *miR-21* and *miR-96* expression levels were normalized against U6 small nuclear RNA (snRNA) expression. Primers used in the study are summarized in Supplementary Table 1.

### Immunoblotting

Whole cell protein extracts were prepared by adding boiling lysis buffer (10 mM Tris-HCl, 1% SDS, 0.2 mM phenylmethylsulfonyl fluoride) including protease and phosphatase inhibitors (Sigma-Aldrich, St Louis, MO) to the cells. Whole tissue protein lysates were prepared by adding boiling lysis buffer, containing protease and phosphatase inhibitors, directly to the tissues, followed by quick homogenization. Both whole cell and tissue lysates were then boiled for 10 min, centrifuged for 35 min, and the supernatants were collected into new tubes. Whole tissue lysates were further centrifuged for 20 min and supernatants were collected into new tubes. All samples were stored at −80 °C. Protein concentrations were measured using NanoDrop 2000c (Thermo Scientific). Equal amounts of protein were loaded onto the wells of Mini-Protean TGX^TM^ 4–15% gels (BioRad Laboratories), and subjected to electrophoresis under reducing conditions. Gels were blotted to nitrocellulose membranes (Millipore, Billerica, MA), which were blocked with 3% bovine serum albumin–1× Tris-buffered saline (TBS) buffer for 1 h at room temperature. Primary antibodies polyclonal rabbit anti-BMPR2 (1:5000; Proteintech, Rosemont, IL #19087-1-AP), monoclonal mouse anti-BMPR2 (1:250, BD Biosciences, San Jose, CA, #612292), monoclonal rabbit anti-RAD51(46B10) (1:1000; Cell Signaling, Danvers, MA #8875), monoclonal rabbit anti-RAD51 [EPR4030(3) (1:5000–9000; Abcam, Burlingame, CA, #ab133534), monoclonal mouse anti-phospho-Histone H2A.X (Ser139), clone JBW301 (1:1000; Millipore, #05-636), polyclonal rabbit anti-p53 (1:500, Cell Signaling, #9282 S), polyclonal rabbit anti-phospho-SMAD1 (Ser463/465)/SMAD5 (Ser463/465)/SMAD9 (Ser465/467) (1:1000, Cell Signaling, #9511), monoclonal mouse anti-SMAD1 (1:1000; Bio Matrix Research. Inc. Noda-shi, Chiba, Japan, #BMR00479), polyclonal rabbit anti-SMAD5 (1:1000, Cell Signaling, #9517), monoclonal mouse GAPDH (1:3000, Millipore, #MAB374), and α-tubulin (clone DM1A, Sigma-Aldrich) were incubated overnight at 4 °C. Before adding secondary horseradish peroxidase-conjugated anti-mouse or anti-rabbit antibodies (1:1000; Cell Signaling, #7074 and #7076, respectively), membranes were washed with 1× TBS or 1× TBST (TBS with Tween-20) buffer. Protein visualization was performed using ECL (Super Signal West Dura, Extended Duration Substrate, Thermo Scientific, #34076). Densitometry was performed to quantify protein amount per sample using ImageJ software (NIH, Bethesda, MD). Normalization was performed against GAPDH protein, and once with α-tubulin.

### Alkaline comet assay

Alkaline comet assay kit was purchased from Trevigen (Trevigen Inc., Gaithersburg, MD) and assay was done following the protocol provided by the manufacturer. In brief, pulmonary microvascular endothelial cells transfected with si-Control, si-BMPR2, or si-RAD51, treated with or without mitomycin C (50 µg/mL; 14 h) or camptothecin (4 µM; 6 h), or pulmonary microvascular endothelial cells treated with LDN193189 (100 nM) for 72 h, or with *miR-96* mimics were washed once with ice-cold 1× phosphate-buffered saline (PBS), de-attached, and briefly centrifuged, before being suspended at 2 × 10^5^ cells/mL in ice-cold 1× PBS. A 50 µL cell suspension was combined with 500 µL LMAgarose, spread onto CometSlide^TM^, and placed at 4 °C in the dark for 10 min. Slides were immersed in Lysis Solution for 60 min at room temperature. Next slides were immersed in Alkaline Unwinding Solution at room temperature for 30 min in the dark. Electrophoresis was performed in Alkaline Electrophoresis Solution (1 V/cm, 300 mA) for 35 min at 4 °C. Slides were immersed in dH_2_O twice for 5 min and then in 70% ethanol for 5 min in the dark at room temperature. Samples were dried at 37 °C for 10–15 min before staining with diluted SYBR® Gold for 30 min. Slides were viewed using upright laser scanning confocal Microscope (LEICA SPE, Buffalo Grove, IL) and the percentage (%) of cells with DNA damage was analyzed by ImageJ software.

### Animal studies

All of the procedures in this study involving vertebrate animals are contained in protocols that have been reviewed by the Institutional Animal Care and Use committee at the University of California, San Francisco (UCSF). The study was performed according to the recommendations in the Guide for the Care and Use of Laboratory Animals of the National Institute of Health. All animals in this study were handled according to institutional animal care and use committee protocols approved by the Committee on the Ethics of Animal Experiments of UCSF and the UK Animals (Scientific Procedures) Act 1986 proved by Home Office Project License 80/2460. For all animal works, determination of group sizes and randomization was done as previously reported^30^. In brief, determination of animal group sizes was done using variance estimation and minimum detectable differences between each group, as previously done based on past experience with pulmonary arterial hypertension rodent models^30^. To perform studies in an unbiased manner^74^, randomization was done using an assigned identification number in each animal, which allowed blinded cardiopulmonary phenotypic procedures, as previously reported^30^.

### Animal models of pulmonary arterial hypertension

#### *BMPR2*^*+*^*/*^*R889X*^ mice

Creation, phenotyping, and sample preparation of *BMPR2*^+^/^R889X^ mice has been previously described^30^. Mice used in the study were all 6 months old with C57Bl/6 background and both male and female mice were studied. *BMPR2*^+^/^R889X^ mice, which showed significantly reduced BMPR2^30^, develop an age-related pulmonary arterial hypertension, which was confirmed by measuring right and left ventricular pressures and volumes with Millar PVR-1045 catheter (Millar Instruments, Houston, TX) and by determining right ventricular hypertrophy using the ratio of right ventricular to left ventricular and septal weight as previously described^30^. *BMPR2*^+/+^ littermates were used as a control.

### Sugen–Hypoxia rat model

Treatment and characterization of rats with SUGEN-5416–hypoxia-induced pulmonary arterial hypertension has been previously described^30^. Lung tissue sample preparation was done as previously described^30^. In brief, male Sprague-Dawley rats (150–200 g, Charles River, UK) were randomly selected to five groups and given a single SUGEN-5416 injection (20 mg per kg, Tocris, Bristol, UK) in a vehicle containing 0.5% carboxyl methylcellulose sodium, 0.4% polysorbate 80, 0.9% benzyl alcohol (Sigma-Aldrich). Rats in group 1 were then placed into a 10% O_2_ chamber and kept in hypoxia for 3 days, removed to normoxia, and killed the following day. Rats in group 2 were placed into a 10% O_2_ chamber for 1 week following normoxia and were killed the following day. Rats in group 3 were placed in hypoxic environment for 3 weeks following the normoxia for further 2 weeks before they were killed. The group 4 rats were placed on hypoxic environment for 3 weeks and kept in normoxia for further 5 weeks to develop pulmonary arterial hypertension. The group 5 rats were used as a control and were given a vehicle (0.5% carboxyl methylcellulose sodium, 0.4% polysorbate 80, 0.9% benzyl alcohol, Sigma-Aldrich). At the each time-point, rats were assessed for cardiopulmonary function and killed as previously described^30,74^.

### Human tissue samples

Human lung tissues were obtained from informed and consenting patients undergoing lung transplantation for end-stage pulmonary arterial hypertension at Royal Papworth Hospital NHS Foundation Trust. Control tissues were obtained from patients undergoing lobectomy or pneumonectomy for lung cancer with tissue being taken from an uninvolved tumor-free region (08/H0304/56+5).

### Immunohistochemistry

Staining of lung sections from normoxia and Sugen–Hypoxia-treated rats for RAD51 detection were performed using the Vector Elite ABC kit (Vector Laboratories, Burlingame, CA) following the manufacturer’s protocol with a rabbit monoclonal RAD51 (EPR4030(3)) (1:200, ab133534, Abcam) and rabbit polyclonal von Willebrand factor (1:100, ab9378, Abcam) antibody. Standard protocol for immunohistochemistry for fluorescence staining was used for γH2AX and von Willebrand factor detection. In brief, sections were deparaffinized with xylene and rehydrated with graded alcohol series followed by heat-induced antigen retrieval using Tris-EDTA buffer (pH 9.0). Quenching of endogenous peroxidase activity was done using 3% H_2_O_2._ Sections were stained using monoclonal mouse anti-phospho-Histone H2A.X (Ser139), clone JBW301 (1:200, Millipore), and rabbit polyclonal von Willebrand factor (1:100, ab9378, Abcam) antibody with overnight incubation at 4 °C. Sections were labeled using secondary antibodies goat anti-mouse IgG, Alexa Fluor plus 555, and goat anti-rabbit IgG, Alexa Fluor 488 (1:250, Thermo Scientific). Lung sections for immunofluorescence were imaged using upright laser scanning confocal microscope Zeiss LSM 780 NLO FLIM with 63× objective and the number of γH2AX-positive endothelial cells were quantitated using ImageJ software (NIH). For human samples, the lung tissue sections were immunostained using a rabbit monoclonal RAD51 (EPR4030(3)) (1:200, Abcam), rabbit polyclonal von Willebrand factor (1:100), and monoclonal mouse anti-mouse/rat/human smooth muscle α-actin (1:100, clone 1A4, Dako, Glostrup, Denmark), labeled using a dextran polymer conjugated secondary antibody, visualized with 3,3’-diaminobenzidine to create a brown reaction product, and counterstained with hematoxylin (all from DakoCytomation, UK). Pulmonary vessels were imaged with light microscope and were quantified and normalized using ImageJ software (NIH).

### Hematoxylin and eosin staining

The hematoxylin and eosin staining was performed following standard procedures^75^. In brief, sections were deparaffinized with xylene and rehydrated with graded alcohol series. Sections were stained with Harris hematoxylin solution followed by counterstain with eosin solution. Sections were dehydrated through graded alcohol series, cleared with xylene, and mounted with xylene-based mounting medium.

### Statistical analysis

Values from multiple experiments are as mean ± SEM. Each individual experiment was repeated at least three times. In all statistical analyses, a normal distribution between samples were assumed. When only two groups were compared, statistical significance was determined using unpaired two-tailed *t*-test and when more than two groups were compared, statistical significance was determined using one-way analysis of variance (ANOVA) followed by Tukey’s multiple comparisons test with 95% confidence interval. A *P* < 0.05 was considered as significant. Prism GraphPad 6 (La Jolla, CA) was used for statistical analyses. The number of samples or animals in each group are indicated in the figure legends.

## Electronic supplementary material

Supplementary Information

## Data Availability

All data generated during this study are included in this published article and its Supplementary Information files.
