# Supplementary material for: Bone morphogenetic protein signaling is required for RAD51-mediated maintenance of genome integrity in vascular endothelial cells
Source: Commun Biol. 2018 Sep 24;1:149. doi: 10.1038/s42003-018-0152-1 (PMC6155317; doi:10.1038/s42003-018-0152-1)
Supplement: Supplementary file 1 — Supplementary Information [file 42003_2018_152_MOESM1_ESM.pdf]

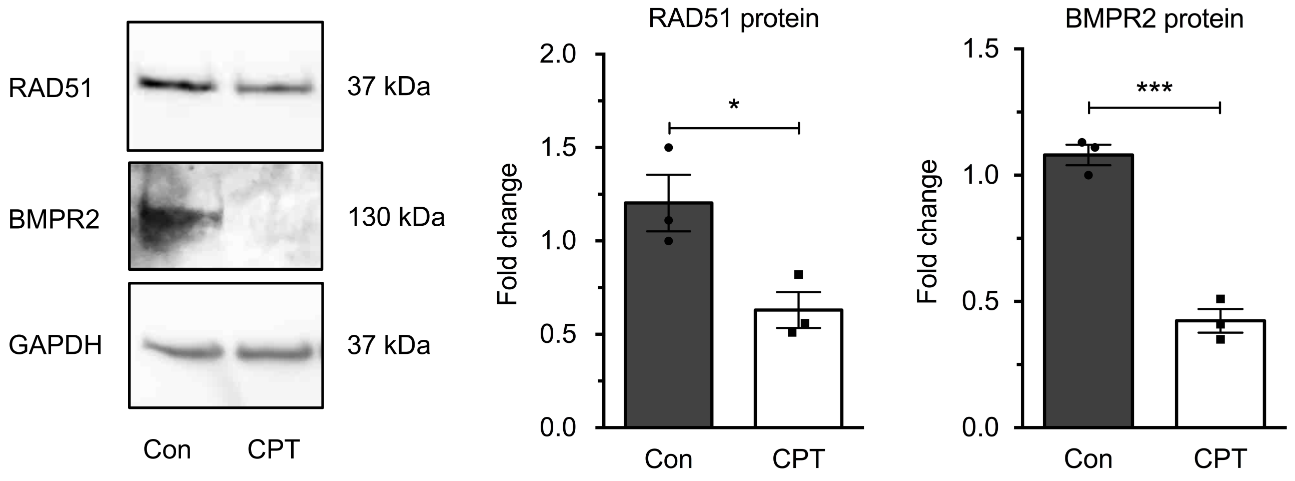

Supplementary Fig. 1. BMPR2 and RAD51 are decreased upon treatment with DNA damage agent Camptothecin. PMVECs were treated with camptothecin (CPT; 4 μM) or vehicle (Con, DMSO) for 6h, followed by immunoblot analysis of RAD51, BMPR2, and GAPDH (loading control). Representative image and the quantitation of three independent blots are shown (n=3). Bars represent mean ±SEM from three different experiments per condition. **P*<0.05 and ****P*<0.01 versus respective control. Unpaired two-tail *t*-test was used.

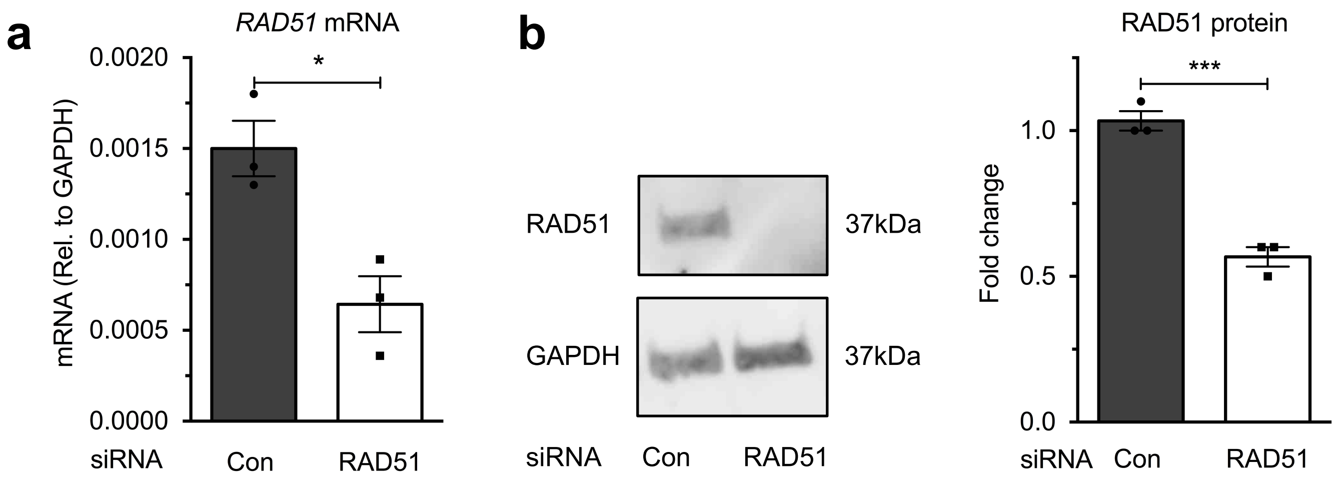

Supplementary Fig. 2. *RAD51* mRNA and protein are efficiently depleted in PMVECs upon siRNA transfection. PMVECs were transfected with control (Con) or *RAD51* siRNAs, followed by qRT-PCR analysis of *RAD51* mRNA (**a**) and immunoblot analysis of RAD51, and GAPDH (loading control) (**b**). Representative image and the quantitation of three independent blots are shown. Bars represent ±SEM from three different experiments per condition (n=3). *P<0.05 and ***P<0.001 versus respective control. Unpaired two-tail *t*-test was used.

Vattulainen-Collanus et al. Supplementary Figure 3

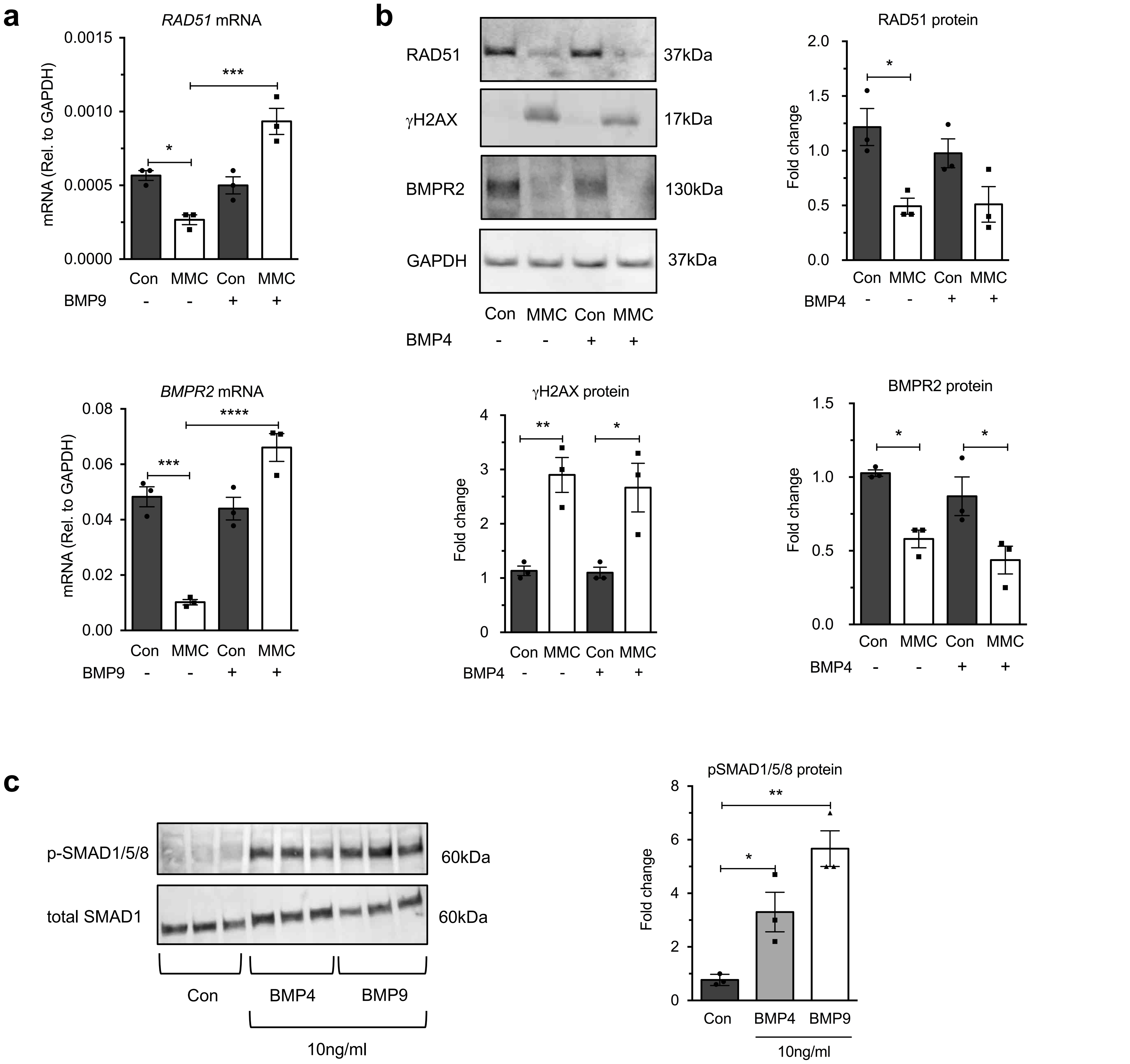

Supplementary Fig. 3. Unlike BMP9, BMP4 stimulation of PMVECs does not rescue the attenuation of RAD51 by Mitomycin C (MMC) treatment. a. PMVECs were treated with vehicle (Con, H_2O) or MMC (50 μg/ml) with or without BMP9 (10 ng/ml) for 14hr. *BMPR2* and *RAD51* mRNA was measured by qPCR (n=3). Normalization was done against *GAPDH* expression. **b.** PMVECs were treated with vehicle (Con, H_2O) or MMC (50 μg/ml) with or without BMP4 (10 ng/ml) for 14hr. RAD51 and BMPR2 protein relative to GAPDH were examined by immunoblot (n=3). Representative image and the quantitation are shown. γH2AX was studied to measure the amount of double strand breaks (DSB). **c.** PMVECs were treated with or without 10 ng/ml BMP4 or BMP9 for 14 hr, followed by immunoblot analysis of phospho-SMAD1/5/8 (p-SMAD1/5/8) and total SMAD1 in triplicates. Bars represent ±SEM from three different experiments per condition (n=3). **P*<0.05, ***P*<0.01, ****P*<0.001, *****P*<0.0001 versus respective control. One-way ANOVA followed by Tukey's multiple comparisons test was used.

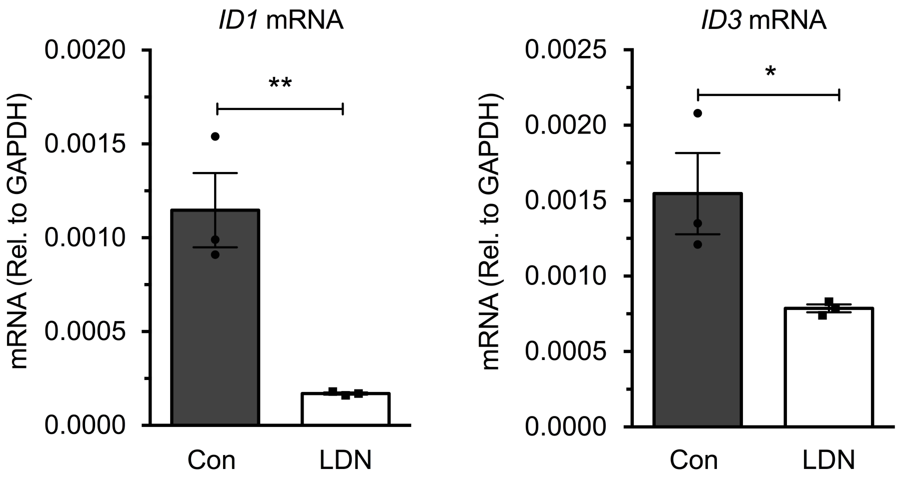

Supplementary Fig. 4. Treatment with the type I BMP receptor kinase inhibitor LDN193189 effectively blocks BMP9-BMPR2-mediated gene regulation. PMVECs were treated with vehicle (Con, DMSO) or LDN193189 (LDN; 100nM) for 72 hr, followed by qRT-PCR analysis of *ID1* and *ID3* mRNA relative to *GAPDH* expression. *ID1* and *ID3* are transcriptionally activated upon BMP stimulation via the BMPR2-SMAD axis. Bars represents ±SEM from three different experiments per condition (n=3). *P<0.05 and **P<0.01 versus respective control. Unpaired two-tail t-test was used.

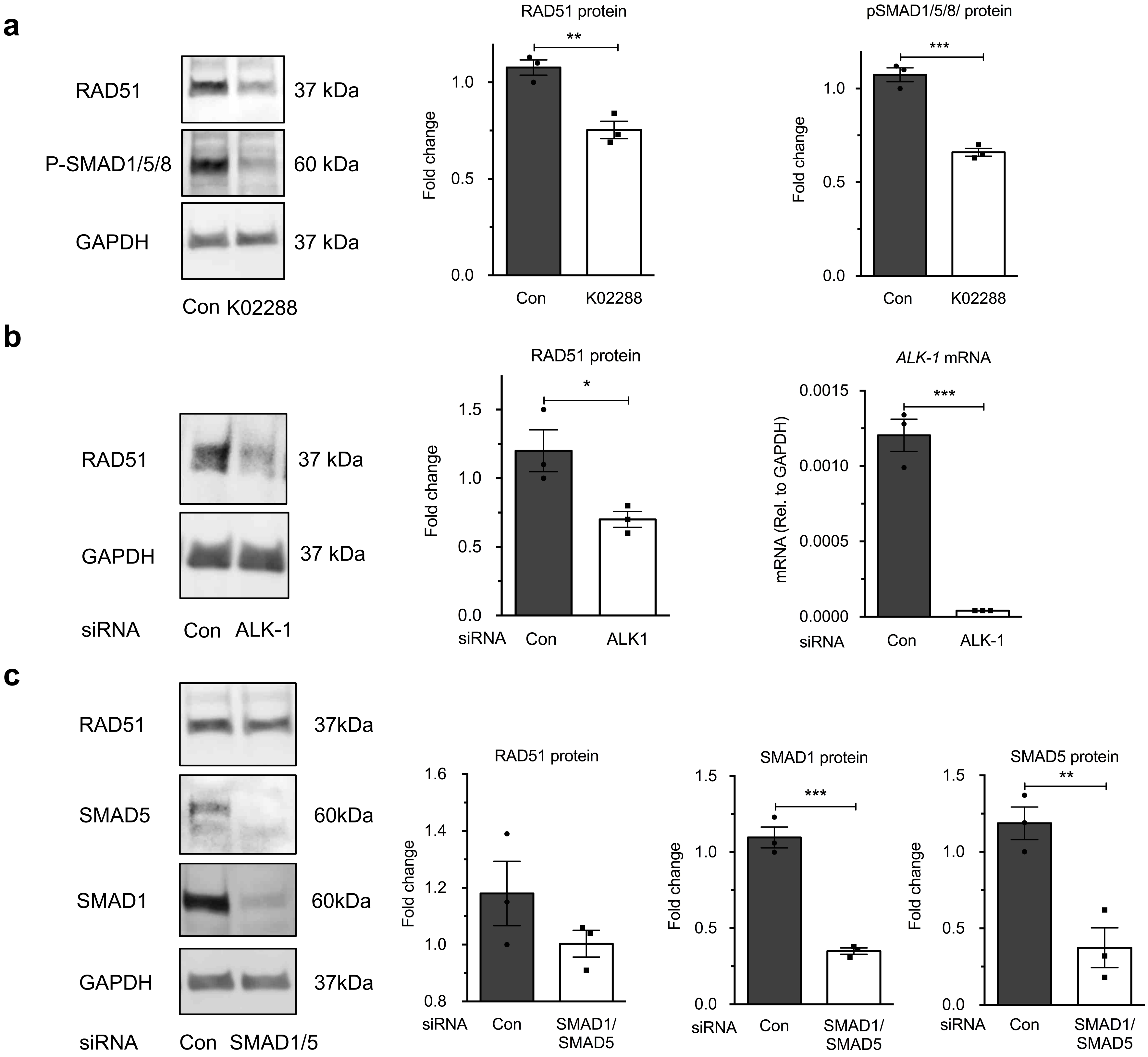

Supplementary Fig. 5. Perturbation of ALK-1 and SMAD1/5 result in RAD51 depletion. a. PMVECs were treated with vehicle (Con, DMSO) or ALK-1/2 inhibitor K02288 (1 μM) for 72 hr, followed by immunoblot analysis of RAD51, phospho-SMAD1/5/8, and GAPDH (loading control). The quantitation of three independent blots (n=3) are shown. **b.** PMVECs were transfected with control (Con) or ALK-1 siRNAs and RAD51 protein amount relative to GAPDH was examined by immunoblot 72 hr after transfections (n=3). The amount of *ALK-1* mRNA relative to *GAPDH* mRNA was determined by qRT-PCR analysis. **c.** PMVECs were transfected with control (Con) or a mixture of siRNAs against SMAD1 and SMAD5. RAD51 protein amount relative to GAPDH was examined by immunoblot 72 hr after transfections (n=3). Bars represent ±SEM from three different experiments per condition. **P*<0.05, ***P*<0.01, and ****P*<0.001 versus respective control. Unpaired two-tail *t*-test was used.

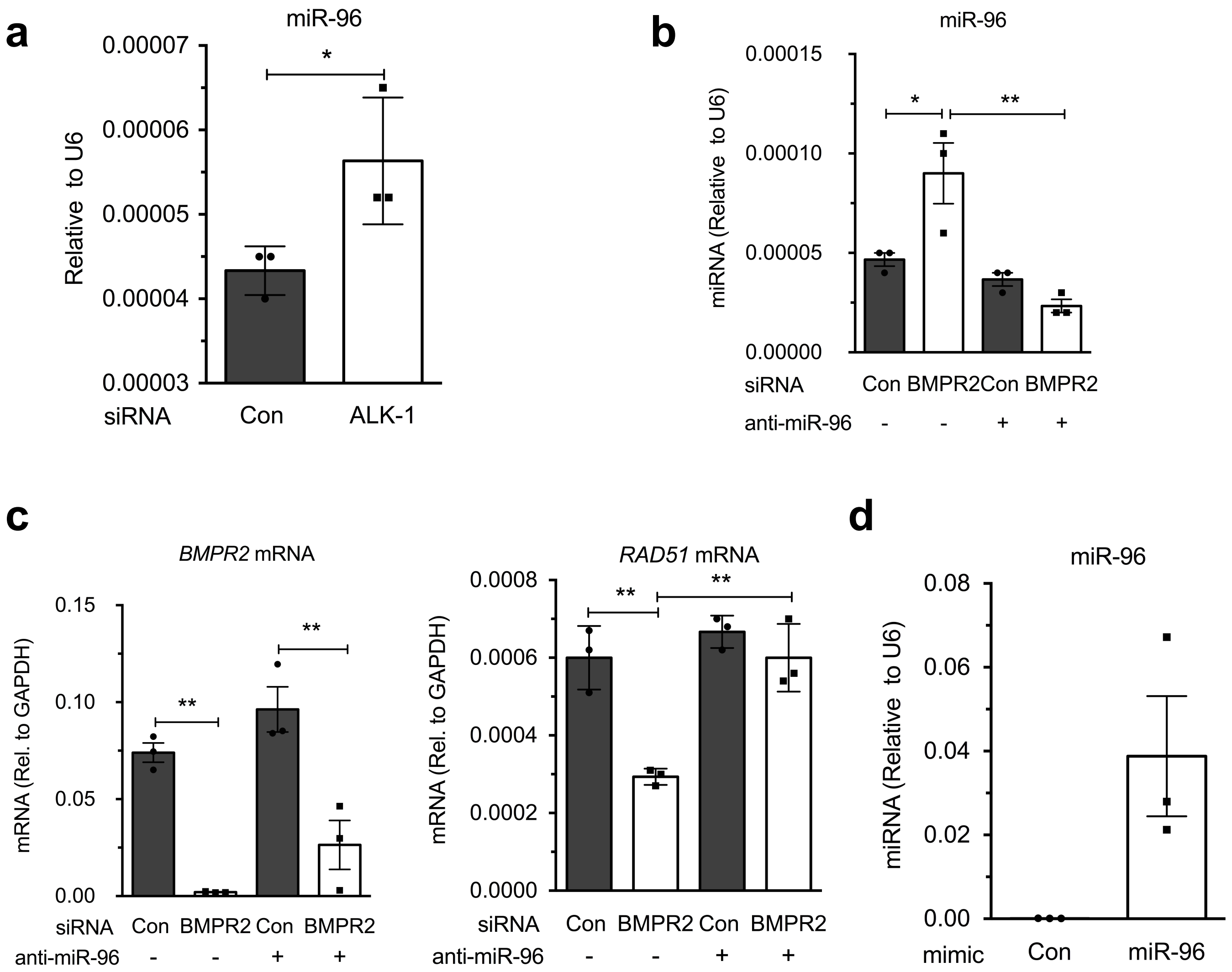

Supplementary Fig. 6. Upregulation of miR-96 expression in PMVECs leads to reduction of RAD51 expression. PMVECs were transfected with control (Con) or ALK-1 siRNAs and miR-96 expression was measured 72h after transfection initiations. Normalization was done against U6 snRNA expression. **a**. PMVECs were co-transfected with control (Con) or BMPR2 siRNAs with anti-Con miRNAs or anti-miR-96 and **b.** miR-96 expression and **c.** *BMPR2* and *RAD51* mRNA expression was measured 48 hr after transfection initiations (n=3). Normalization was done against U6 snRNA and *GAPDH* mRNA expression. **d.** PMVECs were overexpressed with miR-control (Con) or miR-96 mimics and miR-96 expression was measured 48 hr after transfection initiations (n=3). Normalization was done against U6 snRNA expression. Bars represents ±SEM from three different experiments per condition. *P<0.05 and **P<0.01 versus respective control. Unpaired two-tail t-test was used in **a** and **d.** One-way ANOVA followed by Tukey's multiple comparisons test in **b** and **c** was used.

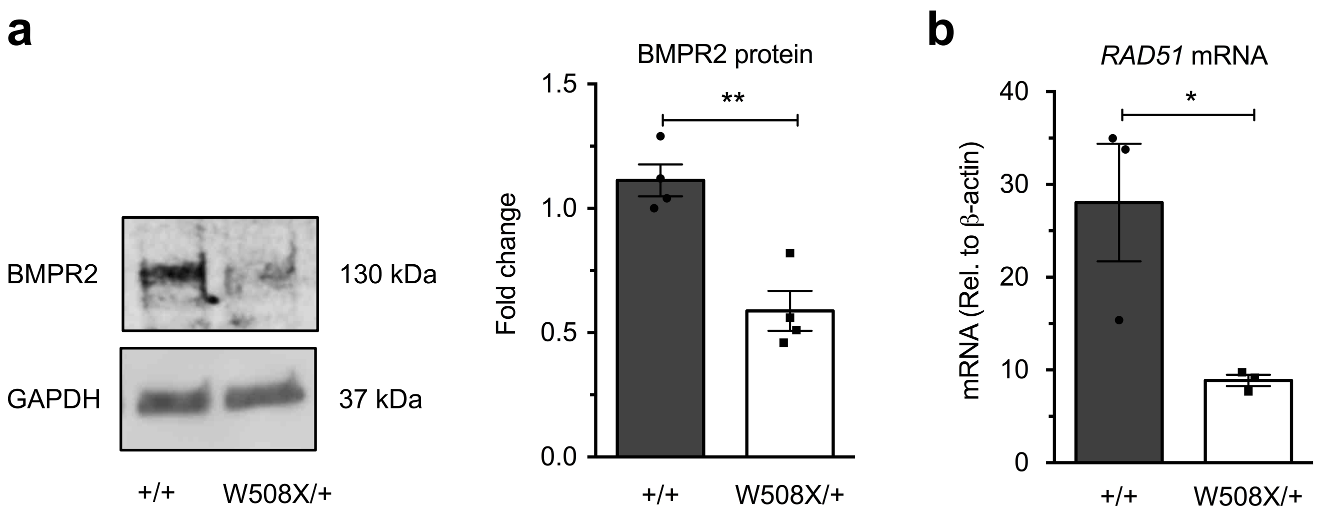

Supplementary Fig. 7. Pulmonary arterial smooth muscle cells (PASMC) isolated from rats with the heterozygous *BMPR2* mutation W508X, leading to reduced BMPR2 amount, show reduced RAD51 expression. a. BMPR2 protein amount and **b.** *RAD51* mRNA expression in wild type (+/+) or W508X/+ rats. Normalization was done against GAPDH expression. Bars represents ±SEM from four different experiments per condition (n=4) in **a** and three different experiments per condition (n=3) in **b**. **P*<0.05 and ***P*<0.01 versus respective control. Unpaired two-tail *t*-test was used.

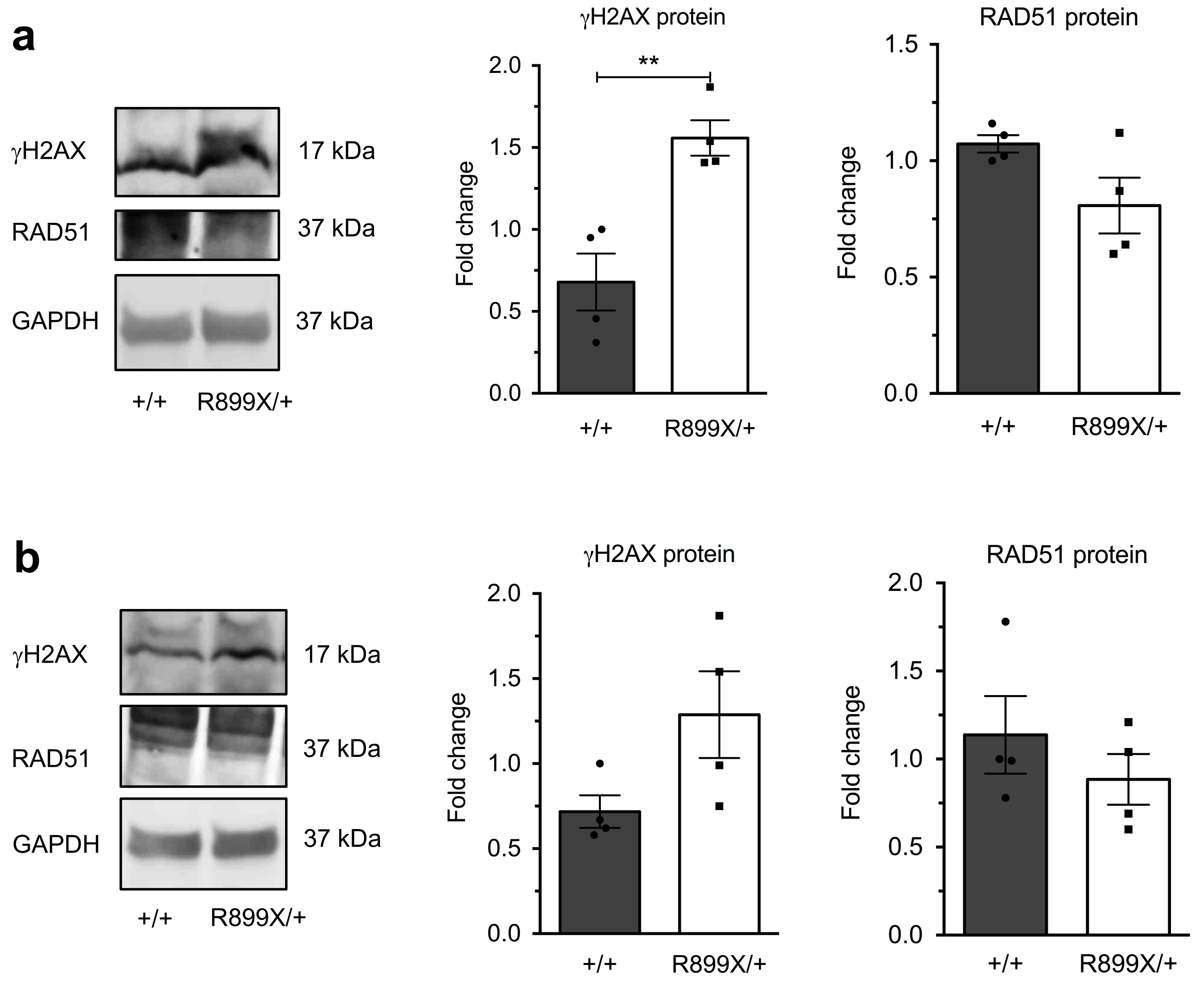

Supplementary Fig. 8. Tissue-specific regulation of RAD51 by BMPR2. The amount of γH2AX, RAD51, and GAPDH (loading control) was examined by immunobloting using total cell lysates of the liver (**a**) and the right ventricle (**b**) from control [BMPR2(+/+)] and heterozygous *BMPR2* mutant (*R899X/+*) mice. Bars represents ±SEM from four different experiments per condition (n=4) in **a** and **b**. Unpaired two-tail *t*-test was used.

Figure 1B

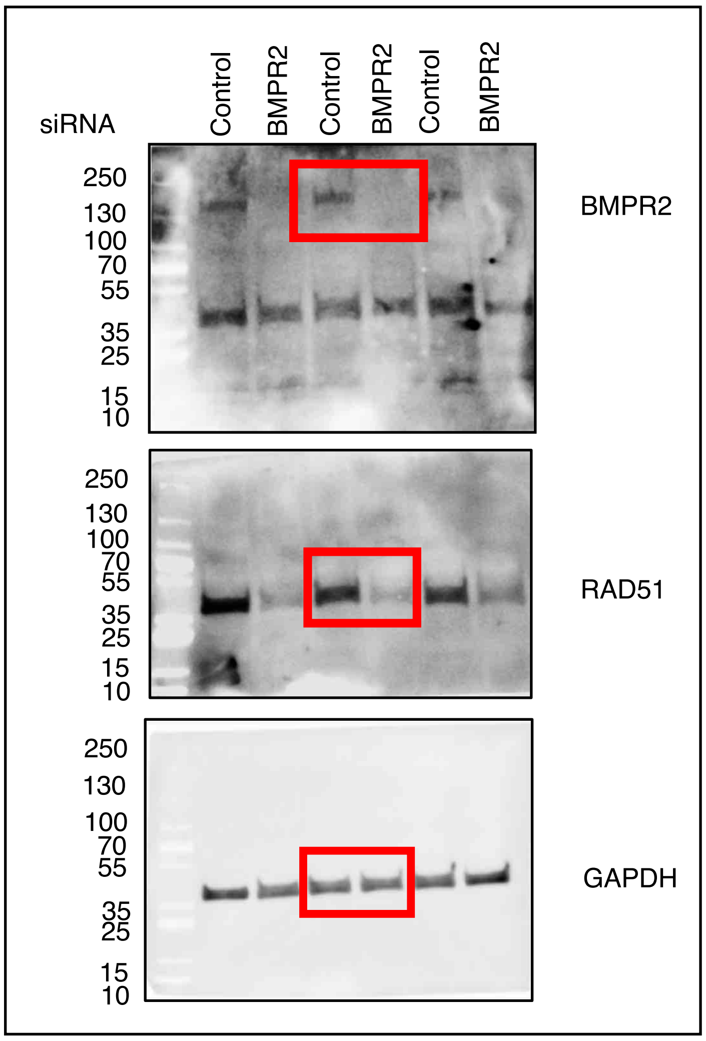

Figure 1D

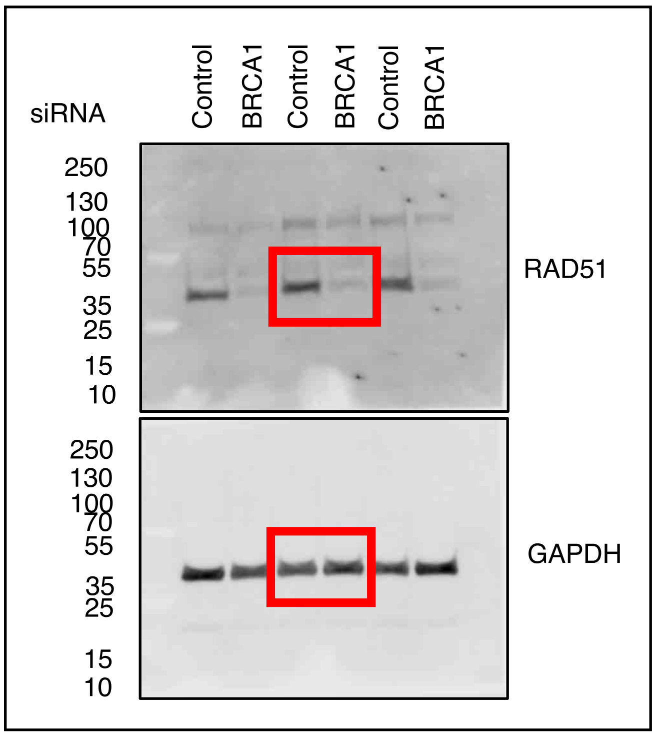

Figure 1F

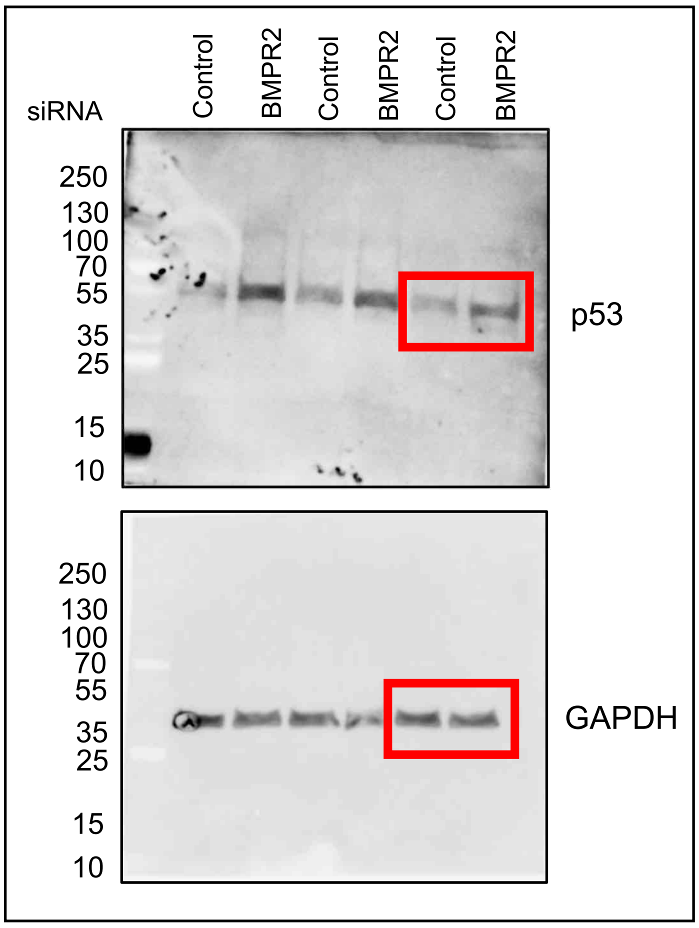

Supplementary Fig. 9. Original immunoblots for Fig. 1.

Figure 2B

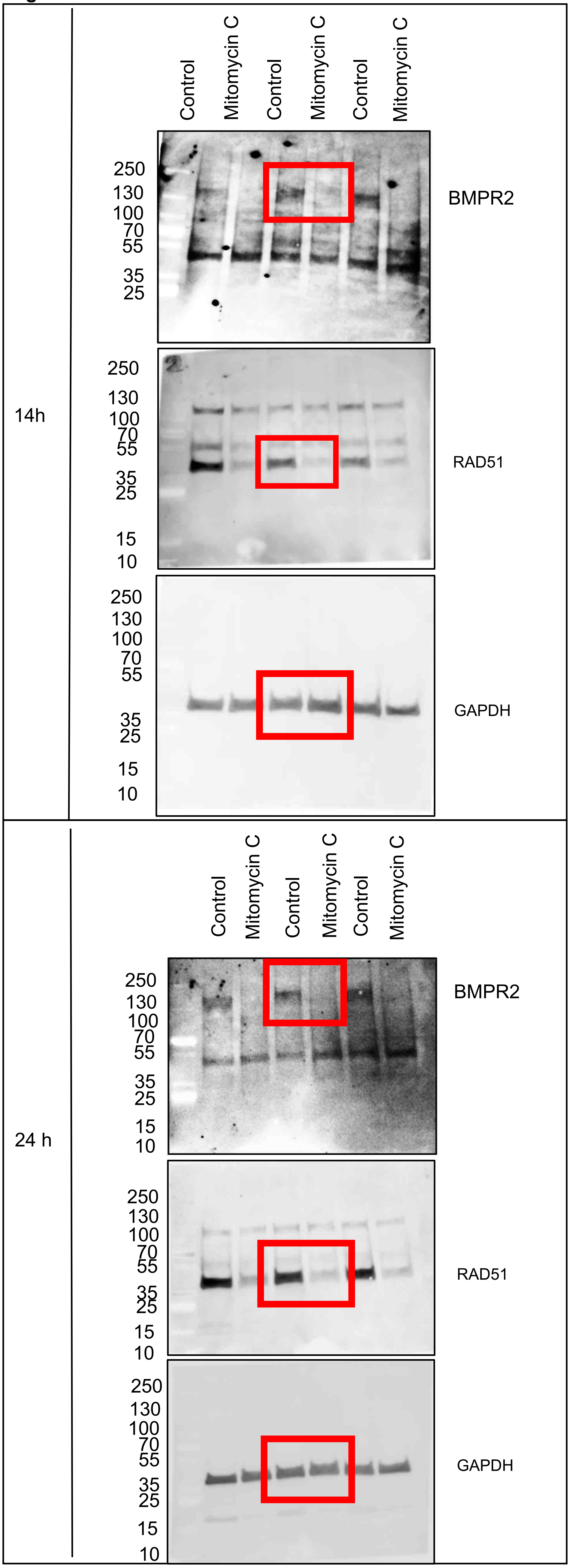

Figure 2C

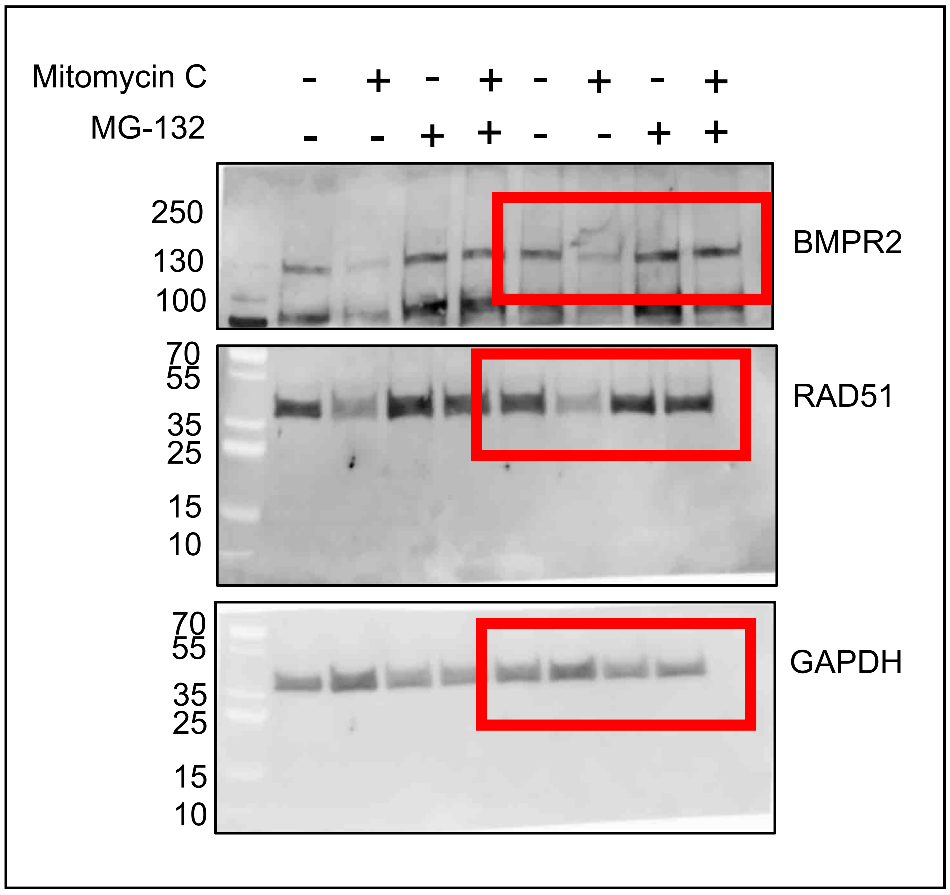

Supplementary Fig. 10. Original immunoblots for Fig. 2.

Figure 3A

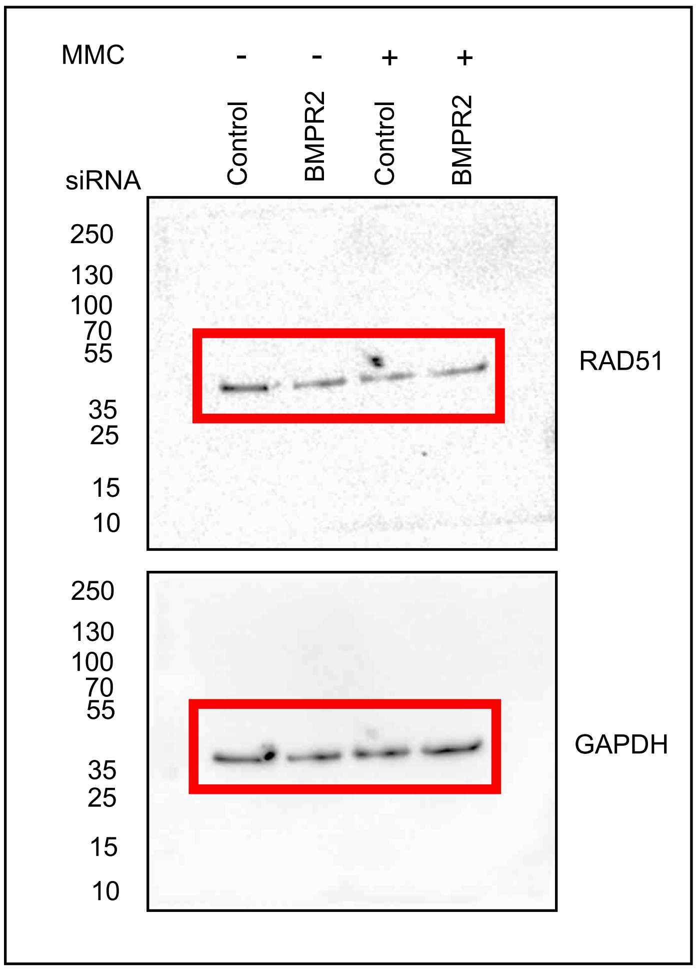

Figure 3E

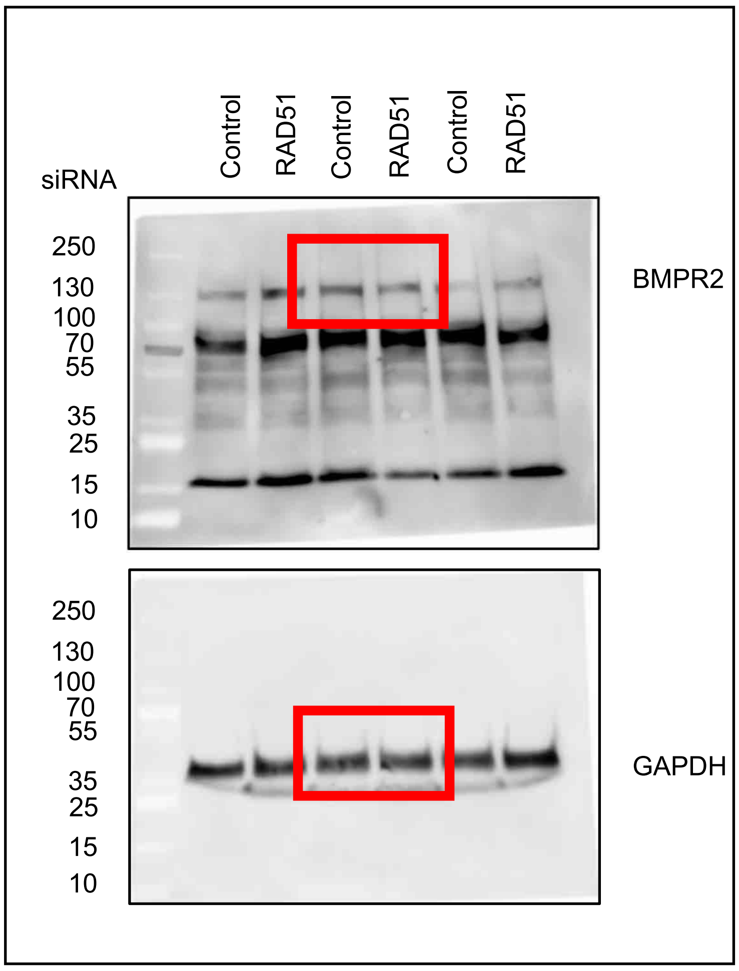

Supplementary Fig. 11. Original immunoblots for Fig. 3.

Figure 4A

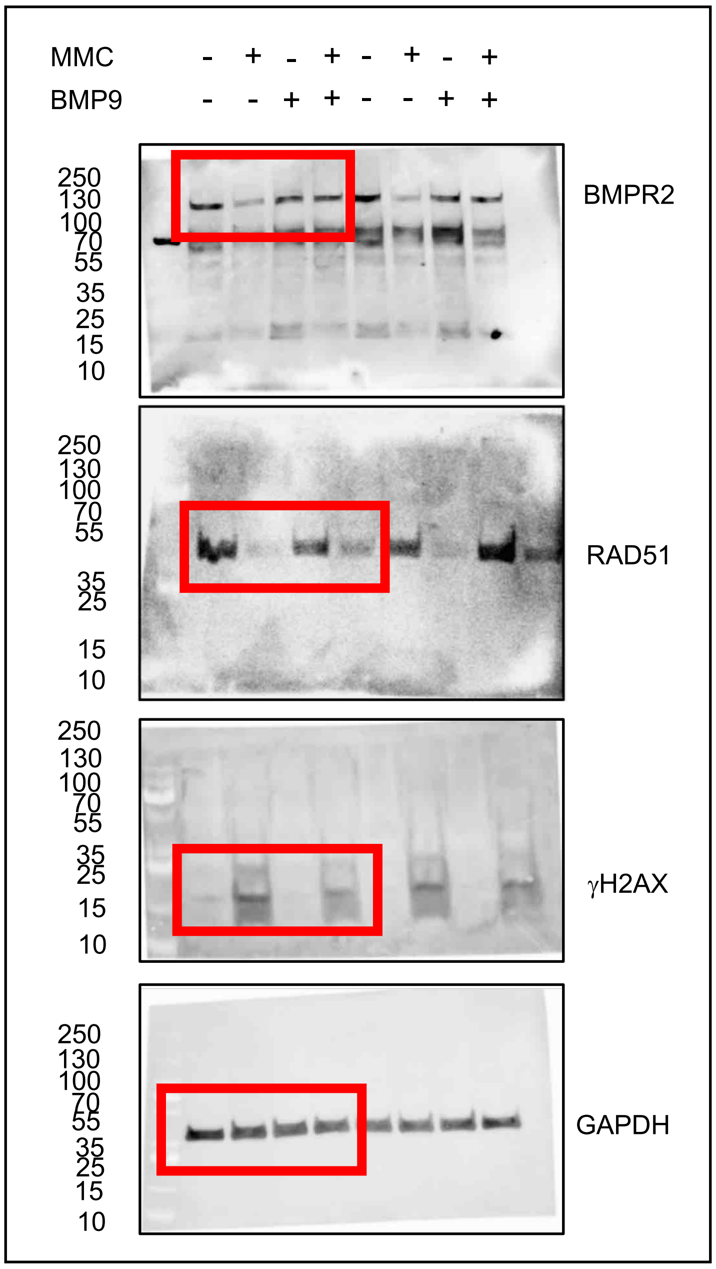

Figure 4B

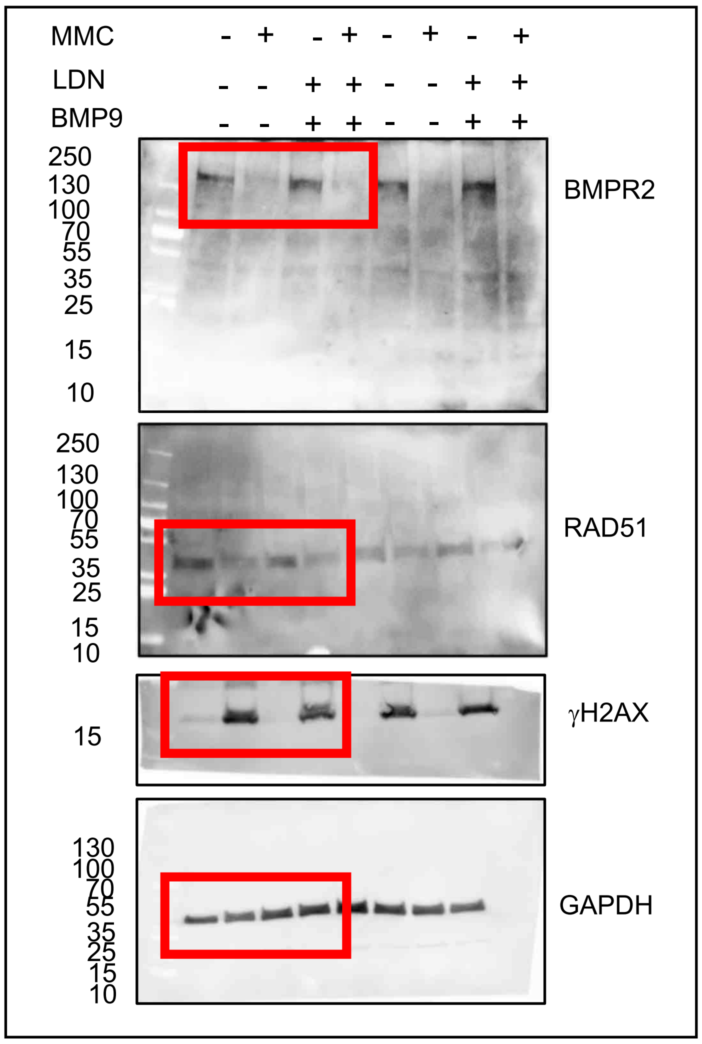

Figure 4C

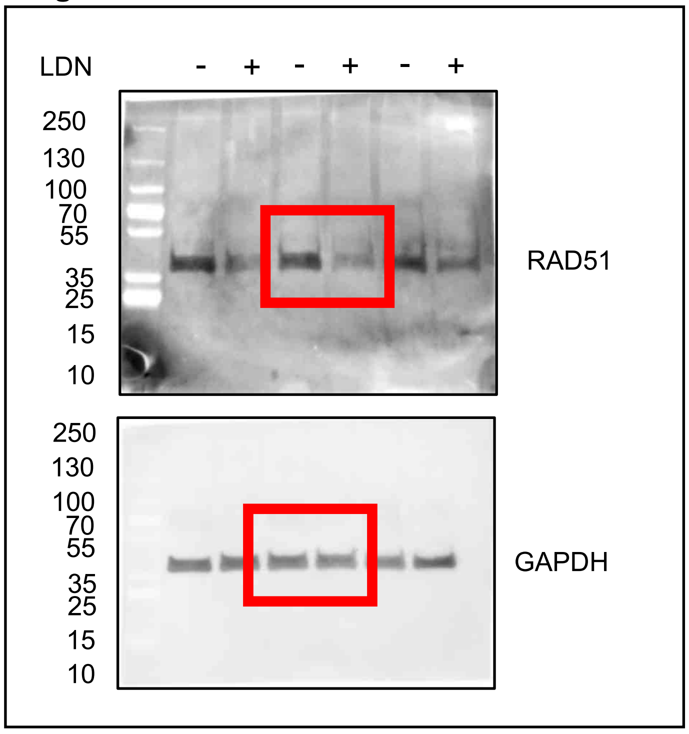

Supplementary Fig. 12. Original immunoblots for Fig. 4.

Figure 5C

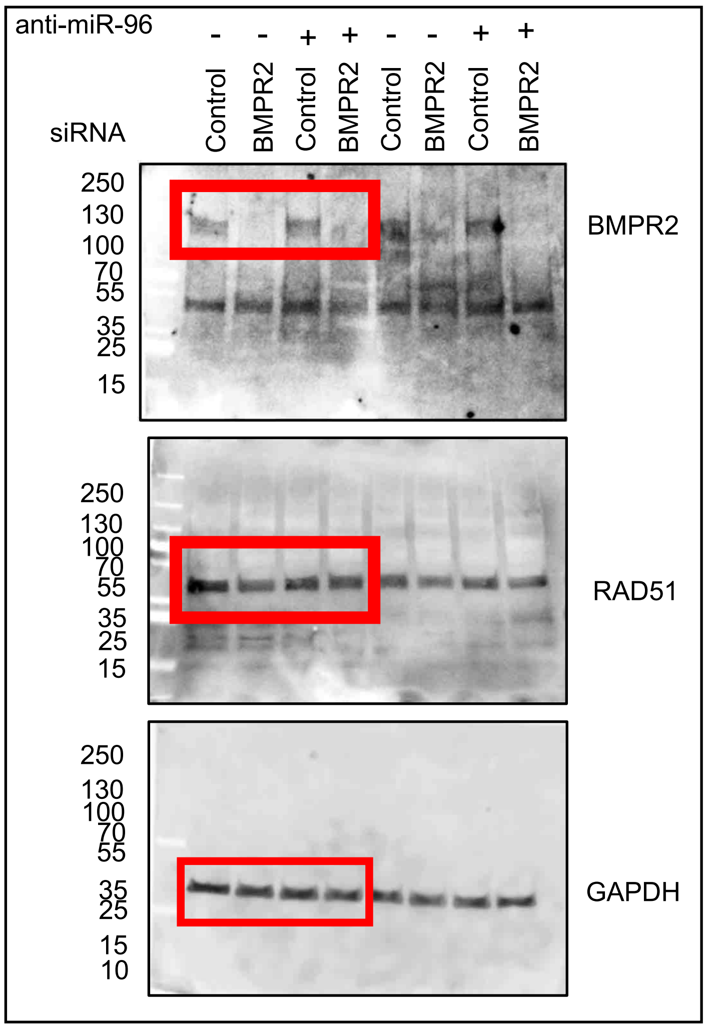

Figure 5D

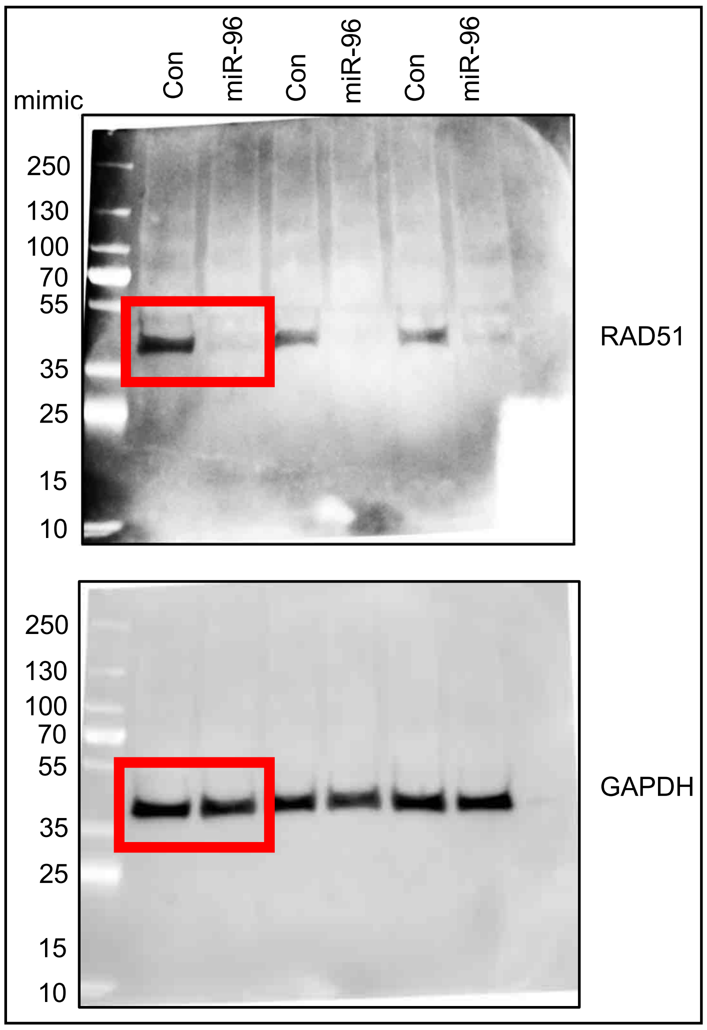

Supplementary Fig. 13. Original immunoblots for Fig. 5.

Figure 6A

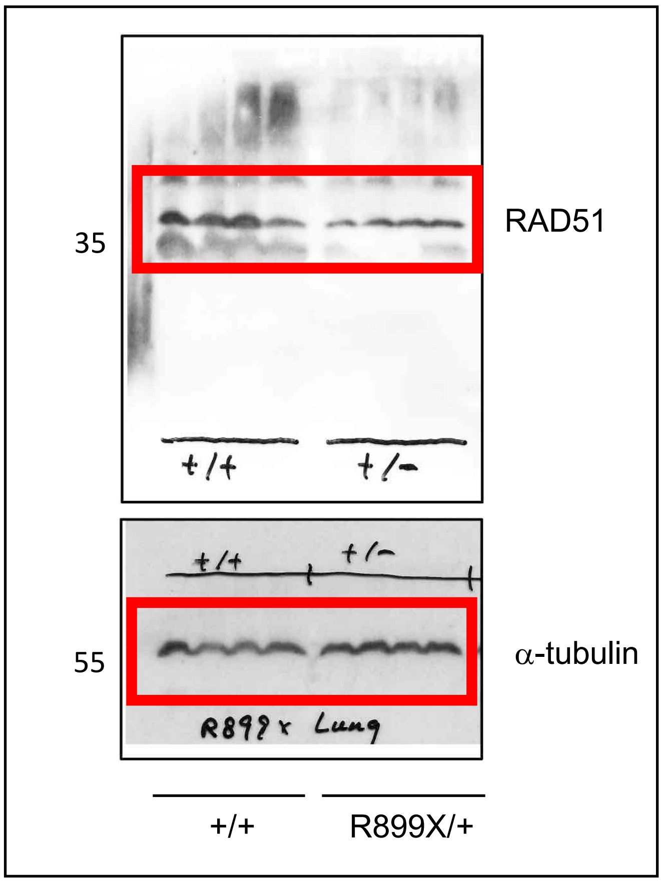

Figure 6B

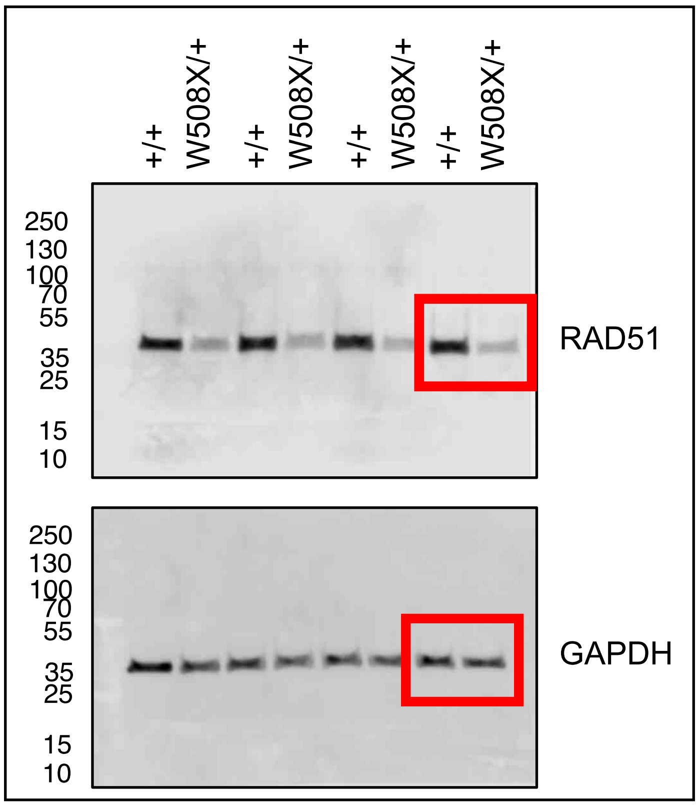

Supplementary Fig. 14. Original immunoblots for Fig. 6.

Supplementary Figure 1

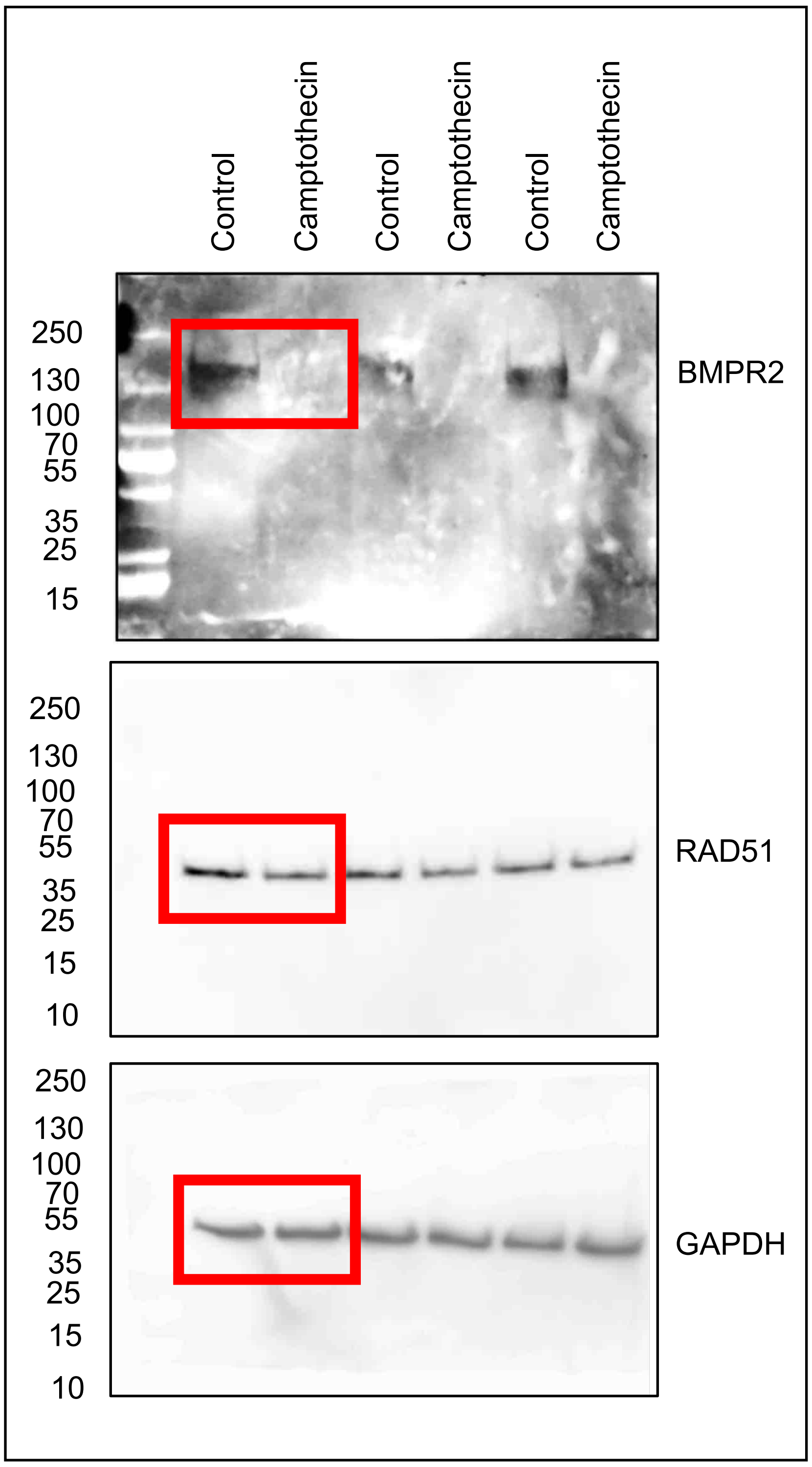

Supplementary Fig. 15. Original immunoblots for Supplementary Fig. 1.

Supplementary Figure 2B.

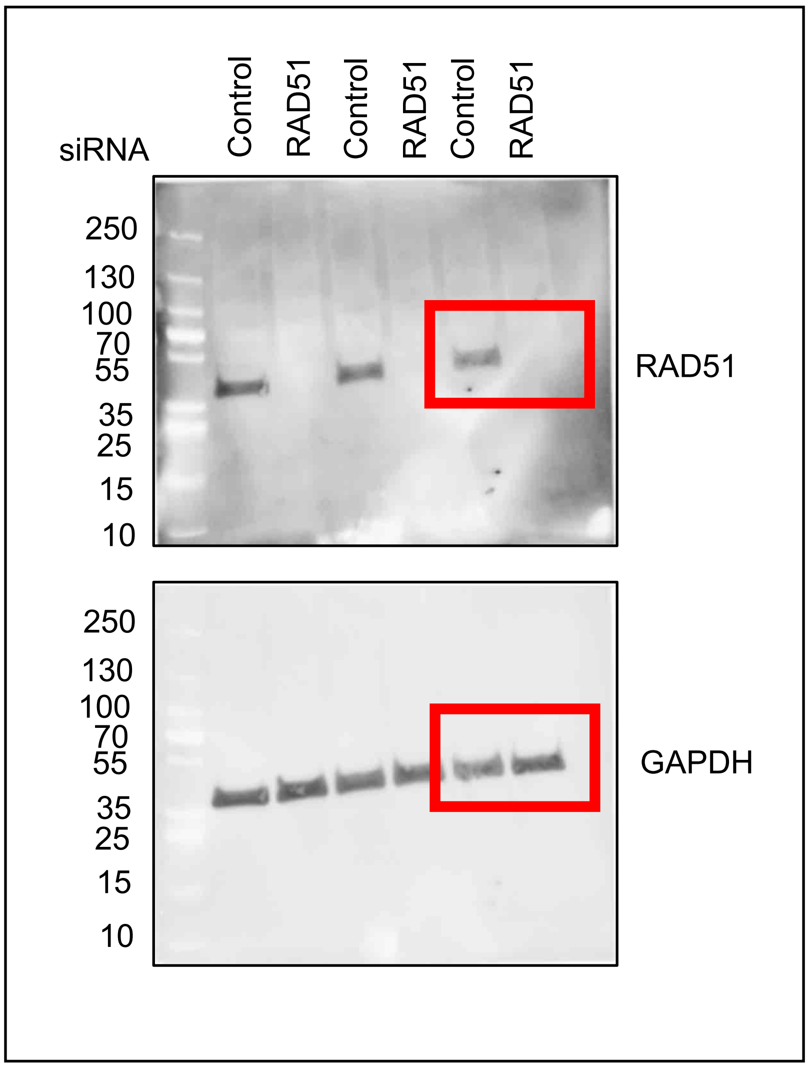

Supplementary Fig. 16. Original immunoblots for Supplementary Fig. 2.

Supplementary Figure 3B

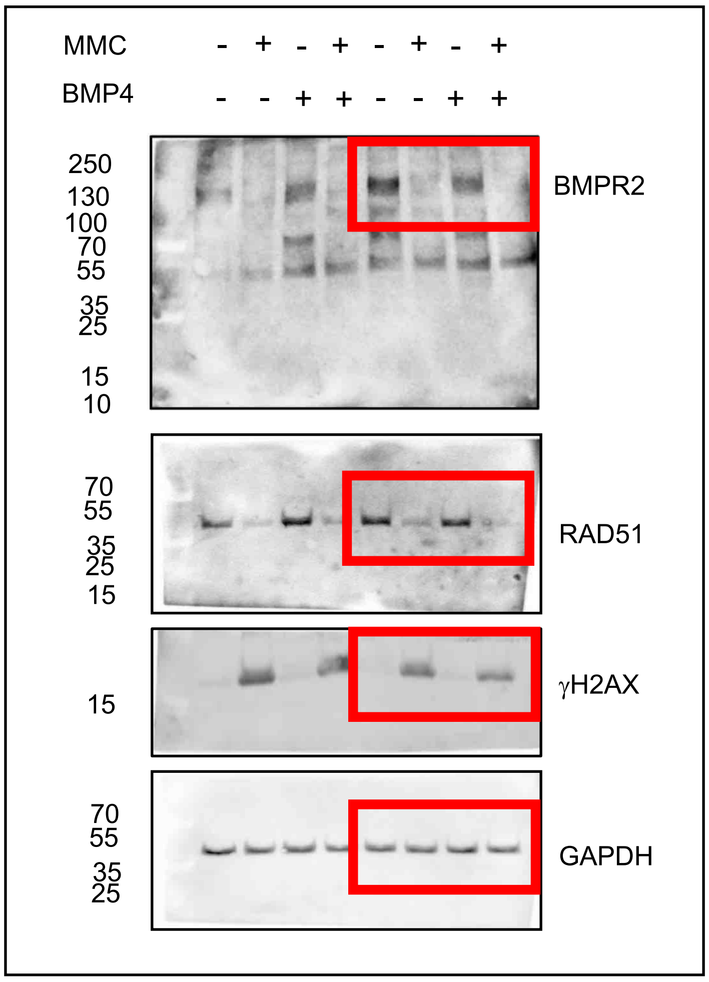

Supplementary Figure 3C

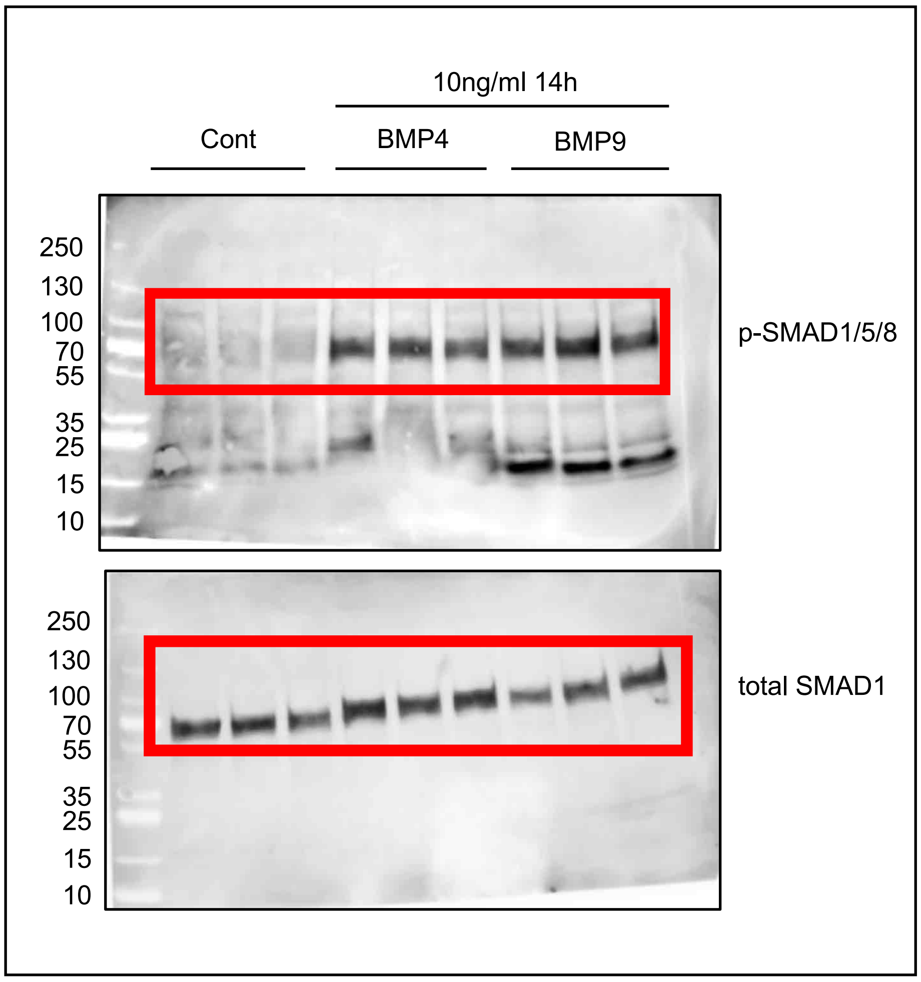

Supplementary Fig. 17. Original immunoblots for Supplementary Fig. 3.

Supplementary Figure 5A

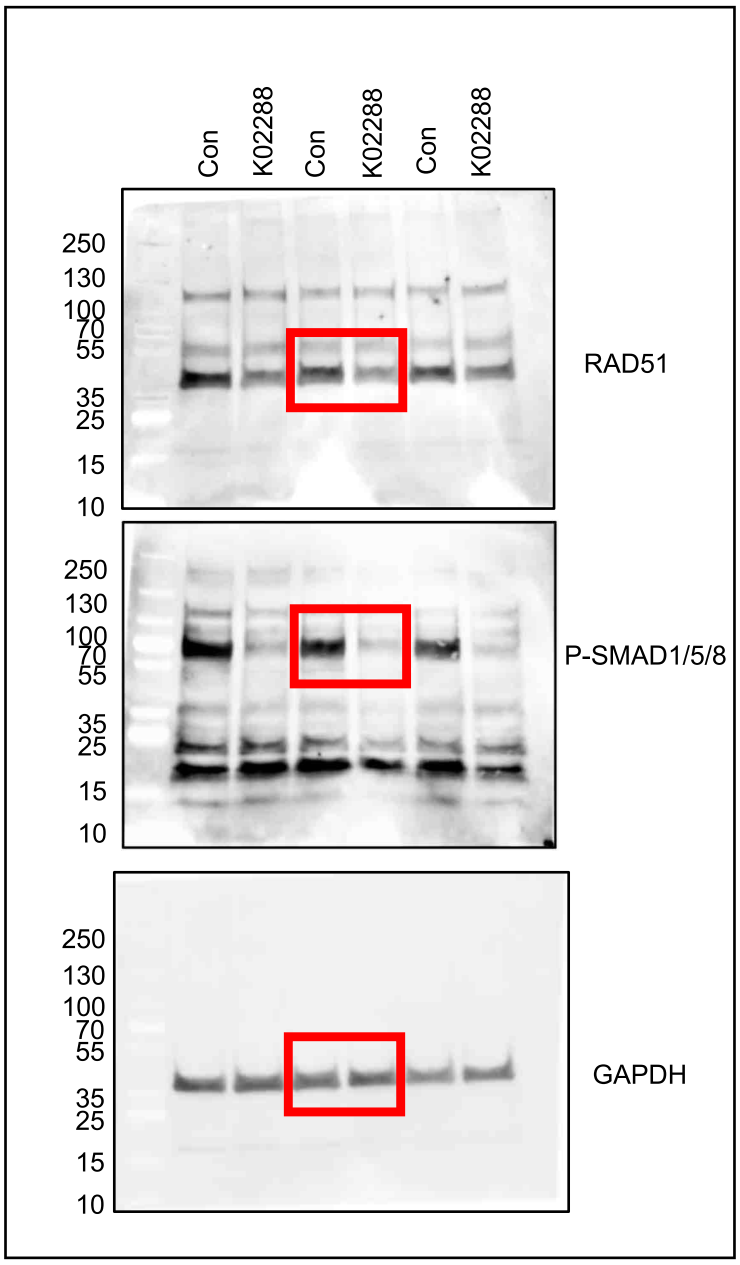

Supplementary Figure 5B

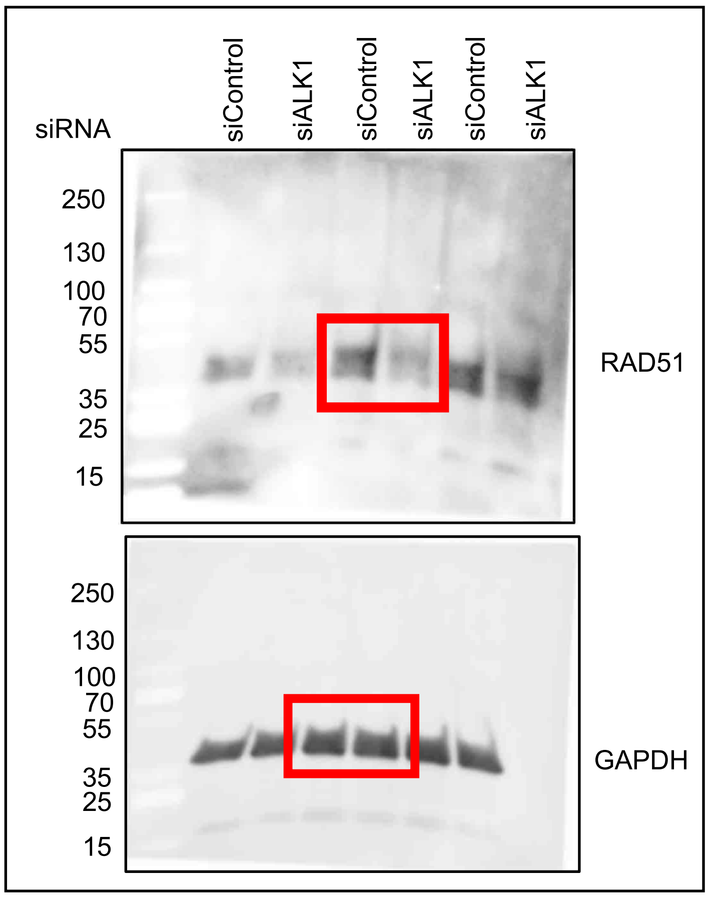

Supplementary Figure 5C

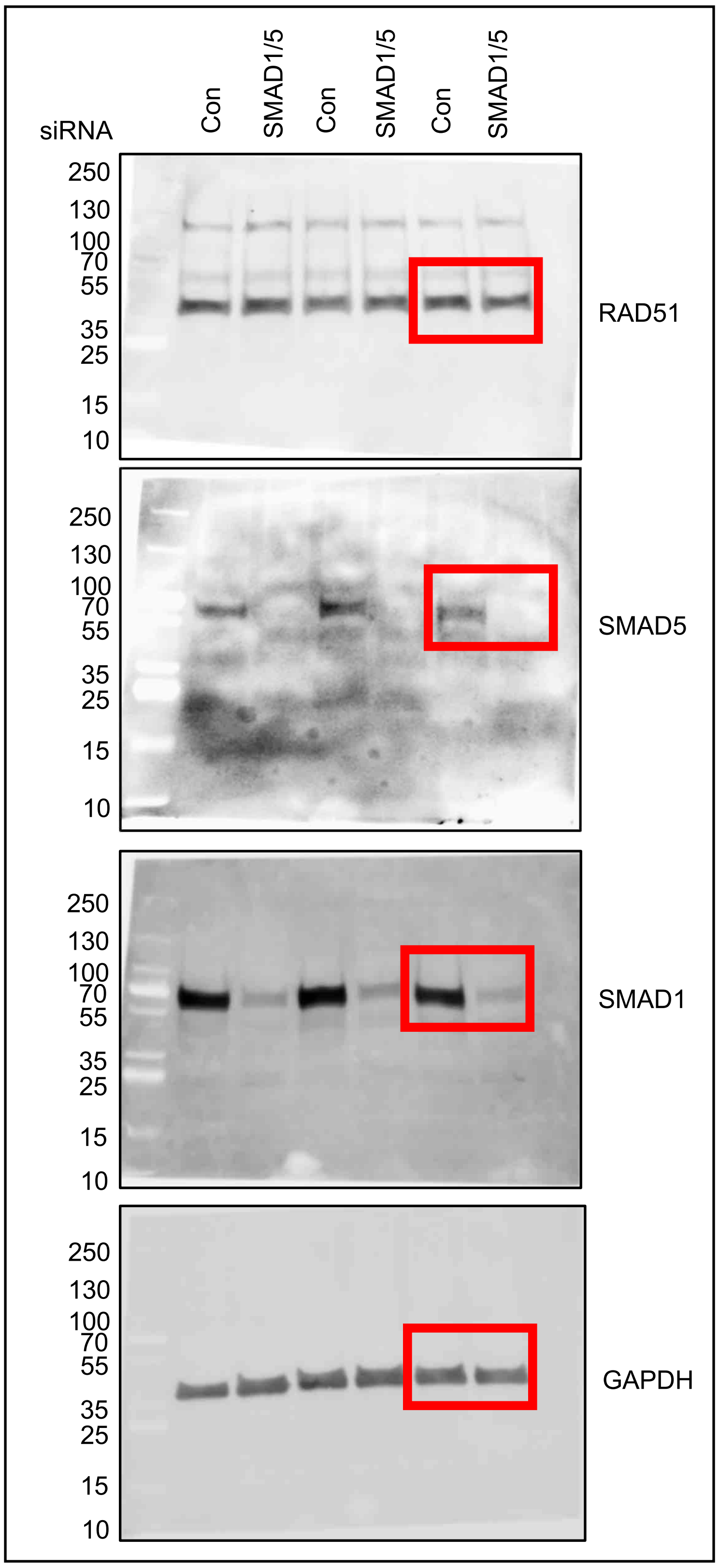

Supplementary Fig. 18. Original immunoblots for Supplementary Fig. 5.

Supplementary Figure 7A

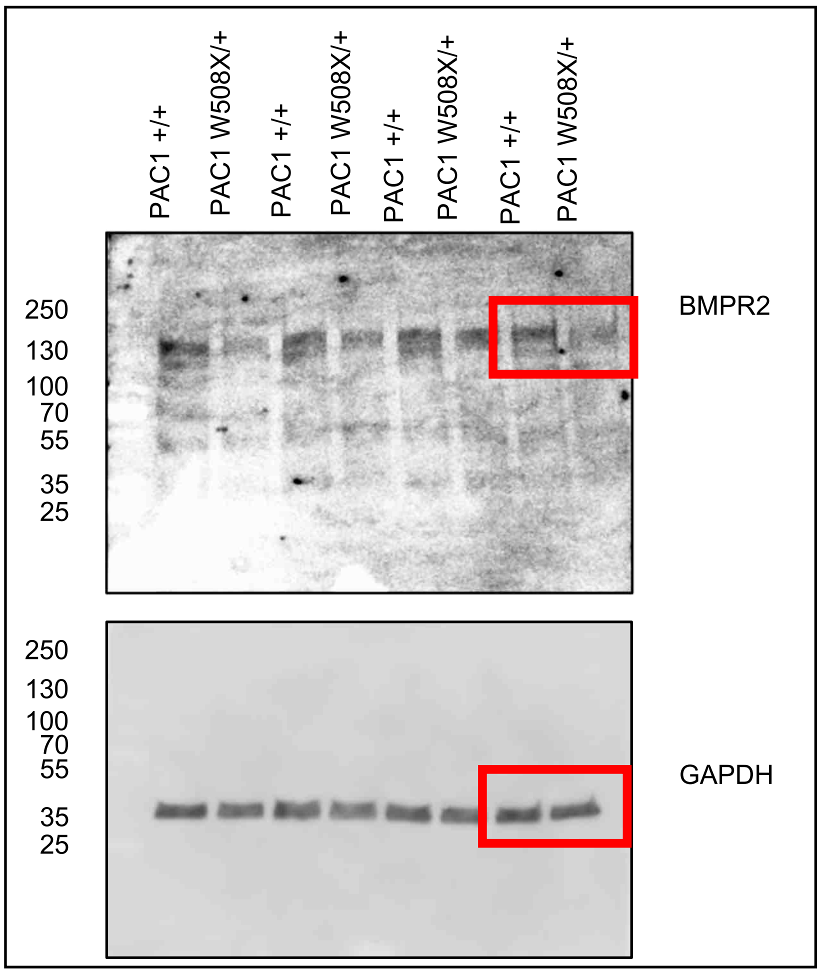

Supplementary Fig. 19. Original immunoblots for Supplementary Fig. 7.

Supplementary Figure 8A

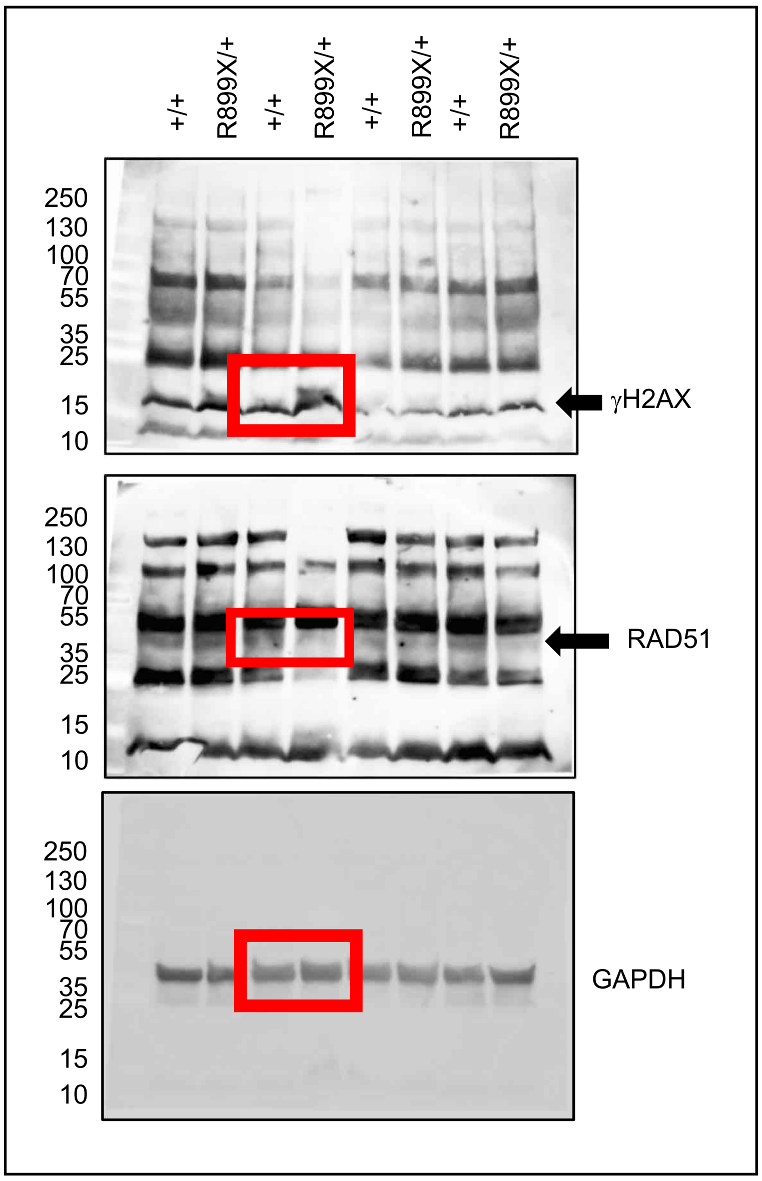

Supplementary Figure 8B

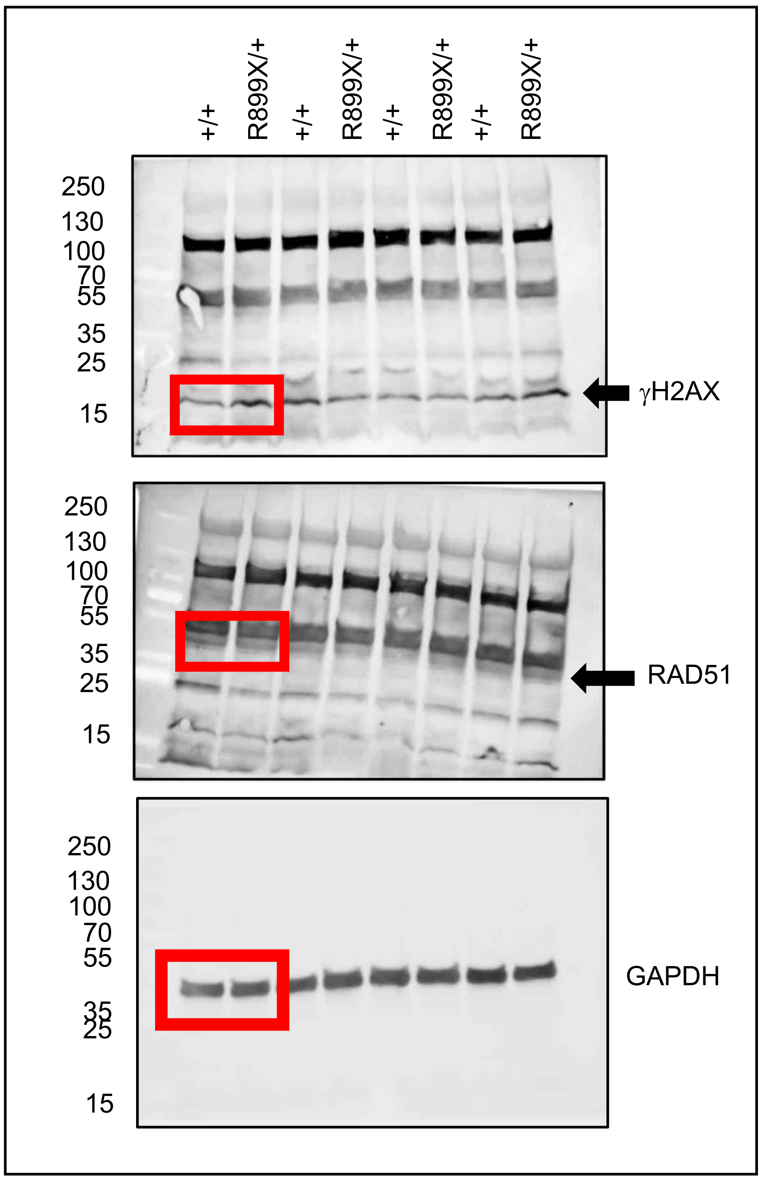

Supplementary Fig. 20. Original immunoblots for Supplementary Fig. 8.

Supplementary Tables

Supplementary Table 1. Primers used in qPCR.

**Gene Symbol**	**Primer Sequence**
human GAPDH	F: 5'-GTCAACGGATTTGGTCGTATTG-3'
	R: 5'-TGGAAGATGGTGATGGGATTT-3'
human BMPR2	F: 5'-GGGTAAGCTCTTGCCGTCTTG-3'
	R: 5'-CCCCTGGGCGCACCAGTCTAT-3'
human BRCA1	F: 5`-CAGTCGGGAAACAAGCATAGA-3`
	R: 5`-AATACTGGAGCCCACTTCATTAG-3`
human RAD51	F: 5`-TGTTGTGACTGCCAGGATAAA-3`
	R: 5`-GGTAGATGGTGAAGGGCTAATG-3`
human p21	F: 5`-GGAAGGGACACACAAGAAGAA-3`
	R: 5`-TCCTTGTTCCGCTGCTAATC-3`
human ID1	F: 5'- GTTGGGAGACTCGCAGGTGT-3'
	R: 5'- CCTGAACCTGCAACAGTTCG-3'
human ID3	F: 5'-GAAAGGTTGCCTGGGACACGCA-3'
	R: 5'-GGCTTTTGTCTGCGCTGTTTTTGTTTC-3'
human ALK-1	F: 5'-GCAACCTGCAGTGTTGCATC-3'
	R: 5'-CGGATCTGCTCGTCCAGCAC-3'
rat beta-actin	F: 5`-GATACTCCCAGCACACTTAAC-3`
	R: 5`-GAAGGGACGAGACTACAACTTAC-3`
rat RAD51	F: 5`-GTGGAATTGAGACTGGGTCTATC-3`
	R: 5`-CTGTCCCACACCTGTCATTT-3`

F=Forward R=Reverse
